# Association of Treatments for Myeloproliferative Neoplasms During Pregnancy With Birth Rates and Maternal Outcomes: A Systematic Review and Meta-analysis

**DOI:** 10.1001/jamanetworkopen.2019.12666

**Published:** 2019-10-04

**Authors:** Dawn Maze, Sajida Kazi, Vikas Gupta, Ann Kinga Malinowski, Rouhi Fazelzad, Prakesh S. Shah, Nadine Shehata

**Affiliations:** 1 Princess Margaret Cancer Center, University Health Network, Toronto, Ontario, Canada; 2 University of Toronto, Toronto, Ontario, Canada; 3 Division of Hematology, University Health Network, Toronto, Ontario, Canada; 4 Division of Maternal–Fetal Medicine, Department of Obstetrics & Gynaecology, Mount Sinai Hospital, Toronto, Ontario, Canada; 5 Department of Medicine, Mount Sinai Hospital, Toronto, Ontario, Canada

## Abstract

**Importance:**

Myeloproliferative neoplasms (MPNs) are increasingly being identified in women of childbearing potential. Pregnancy in women with MPNs is associated with maternal thrombosis, hemorrhage, and placental dysfunction leading to fetal growth restriction or loss.

**Objective:**

To evaluate the association between the use of aspirin, heparin, interferon, or combinations and live birth rate and adverse maternal outcomes in pregnant women with MPNs.

**Data Sources:**

Systematic searches of MEDLINE, Embase, Cochrane Database of Systematic Reviews, Cochrane Central Register of Controlled Trials, and the MEDLINE Epub Ahead of Print and In-Process and Other Non-Indexed Citations from inception to July 19, 2018, with no language restrictions, was conducted. Key search terms included *myeloproliferative disorders*, *polycythemia vera*, *essential thrombocythemia*, and *primary myelofibrosis*.

**Study Selection:**

A study was eligible if it included pregnant patients with MPNs; interventions included aspirin, heparin, and/or interferon; there was a comparison group in which patients did not receive the intervention; the study reported on at least 1 of the study outcomes; and it was a randomized, case-control, or cohort study or series of at least 10 pregnancies. Data were extracted in duplicate; 0.5% of identified studies met selection criteria.

**Data Extraction and Synthesis:**

The review was conducted according to Preferred Reporting Items for Systematic Reviews and Meta-analyses (PRISMA) guidelines and reported in accordance with Meta-analysis of Observational Studies in Epidemiology (MOOSE) guidelines. Data were pooled using the Mantel-Haenszel approach.

**Main Outcomes and Measures:**

Outcomes were the number of live births and maternal complications, specifically, arterial or venous thrombosis, hemorrhage, and preeclampsia.

**Results:**

Twenty-two studies reporting on 1210 pregnancies were included. The live birth rate was 71.3% (95% CI, 65.1%-77.6%). Use of aspirin (11 studies, 227 patients; unadjusted odds ratio, 8.6; 95% CI, 4.0-18.1) and interferon (6 studies, 90 patients; unadjusted odds ratio, 9.7; 95% CI, 2.3-41.0) were associated with higher odds of live birth. Addition of heparin to aspirin was not associated with higher odds of live birth (6 studies, 96 patients; unadjusted odds ratio, 2.1; 95% CI, 0.5-9.0). The most common adverse maternal event was preeclampsia, with an incidence of 3.1% (95% CI, 1.7%-4.5%).

**Conclusions and Relevance:**

Most studies reported on pregnancy with essential thrombocythemia. Few studies reported on pregnancy with polycythemia vera and none with myelofibrosis met the inclusion criteria. Most studies were retrospective and early pregnancy losses may have been underreported. Moderate-quality evidence suggests that aspirin or interferon is associated with higher odds of live birth in pregnant women with MPN.

## Key Points

**Question:**

Are use of aspirin, heparin, interferon, or combinations associated with live birth rate and adverse maternal outcomes in pregnant women with myeloproliferative neoplasms?

**Findings:**

In this systematic review and meta-analysis of 22 studies, reporting on 1210 pregnancies, the live birth rate was 71.3%; most studies reported on pregnancy with essential thrombocythemia. The use of aspirin and interferon—but not heparin—was associated with higher odds of live birth.

**Meaning:**

Moderate-quality evidence suggests that treatment with aspirin or interferon is associated with higher odds of live birth in pregnant patients with myeloproliferative neoplasms.

## Teaser

This systematic review and meta-analysis examines whether the use of aspirin, heparin, and interferon in pregnant women with myeloproliferative neoplasms is associated with live birth rate and adverse maternal outcomes.

## Introduction

Essential thrombocythemia, polycythemia vera, and myelofibrosis are hematopoietic stem cell–derived clonal disorders collectively known as the classic Philadelphia chromosome–negative myeloproliferative neoplasms (MPNs). Although they are a heterogeneous group of disorders, MPNs share common complications, such as thrombotic and hemorrhagic events; debilitating microvascular, constitutional, or other disease-related symptoms; and fibrotic or leukemic transformation.

Although MPN diagnoses are typically made in the sixth or seventh decade of life, approximately 20% of patients with essential thrombocythemia^1^ and 15% of patients with polycythemia vera^2,3^ are younger than 40 years. Furthermore, given the widespread use of automated cell counters and increasing access to mutational analysis, MPNs continue to be diagnosed earlier.^1,4,5^ Combined with the societal trend of advancing maternal age, MPNs are increasingly being identified in women who are pregnant or planning a pregnancy. There is limited information on fetal and maternal outcomes and optimal management of pregnancy in patients with MPNs, with most of the guidance from narrative reviews and expert opinion.^1,5,6,7^ Pregnancy with MPNs has been reported to be associated with maternal thrombosis, hemorrhage, and placental dysfunction leading to fetal growth restriction or loss. To improve outcomes and reduce complications, several strategies have been attempted, including aspirin, heparin, and cytoreduction with interferon. However, results have been variable with, for example, some studies reporting an increase in live birth rate with antepartum aspirin alone or in combination with other agents,^8,9,10^ and others reporting no benefit.^11^ It is also possible that antepartum use of aspirin and/or heparin may increase the risk of maternal bleeding events.

The goal of this systematic review and meta-analysis was to review whether treatment with aspirin, heparin, interferon, or combinations thereof is associated with the live birth rate and adverse maternal outcomes in pregnant patients with MPNs.

## Methods

This study followed the Preferred Reporting Items for Systematic Reviews and Meta-analyses (PRISMA) reporting guideline^12^ and is reported in accordance with the Meta-analysis of Observational Studies in Epidemiology (MOOSE) guidelines.^13^

### Data Sources and Searches

A systematic search was conducted by a librarian (R.F.) using MEDLINE (1946 to July 2018), Embase (1947 to July 2018), Cochrane Database of Systematic Reviews (2005 to July 2018), Cochrane Central Register of Controlled Trials (inception to June 2018), and MEDLINE Epub Ahead of Print and In-Process and Other Non-Indexed Citations (inception to July 2018) (eTable 1 in the Supplement). Key search terms included *myeloproliferative disorders*, *polycythemia vera*, *essential thrombocythemia*, and *primary myelofibrosis*. The searches were completed July 19, 2018. Bibliographic references were reviewed to identify additional studies. There were no date or language restrictions, and translators were used when applicable. Peer-reviewed abstracts and conference proceedings were included if published between January 1, 2013, and July 19, 2018. Where applicable, authors were contacted for clarification or additional information. Citations were reviewed in duplicate.

### Study Selection

A study was eligible for inclusion if (1) participants were pregnant patients with one of the classic Philadelphia chromosome–negative MPNs, essential thrombocythemia, polycythemia vera, or myelofibrosis. Studies of patients with chronic myeloid leukemia, rare MPNs (eg, hypereosinophilias), and myelodysplastic/MPN overlap syndromes were excluded; (2) interventions included at least 1 of aspirin, unfractionated heparin or low-molecular-weight heparin (LMWH), or interferon; and (3) there was a comparison group in which patients did not receive the intervention (ie, treatment was managed with observation alone or different intervention). This latter criterion was required for the meta-analysis only; studies did not need a comparator to be included in the qualitative synthesis. In addition, the study must have reported on at least 1 of the outcomes of interest and it was a randomized clinical trial, cohort study, case-control study, or case series of at least 10 pregnancies. While a minimum of 10 pregnancies were required for study inclusion, the number of pregnancies in intervention groups could be less.

### Data Extraction and Quality Assessment

Data were independently extracted by 2 of us (D.M. and S.K.) using a standardized collection form. Data were compared and discrepancies resolved by consensus. One of us (N.S.) confirmed the data and adjudicated any remaining discrepancies. Duplicate reports were excluded, with only the most recent or most informative study included. Where required, authors were contacted for data clarification. The data extracted included year and setting of publication, type of MPN, driver mutation, number of patients and pregnancies, and treatment details. Outcomes of interest were the number of live births and maternal complications, specifically, arterial or venous thrombosis, hemorrhage (except for epistaxis and mucocutaneous bleeding), and preeclampsia. For the outcome live births, the denominator was the number of fetuses to account for multiple pregnancies.

Risk of bias for individual studies was assessed using the Newcastle-Ottawa Scale.^14^ A study could be awarded 1 star for each of the numbered items with the selection (maximum 4) and outcome (maximum 3) categories. Within the comparability section, a maximum of 1 star could be awarded if the study controlled for prior live births. The criteria were not modified to indicate specific control for a second factor; therefore, the maximum score that a study could receive was 8 (eTable 2 in the Supplement). Studies receiving more than 6 stars were considered to be at low risk of bias, those receiving 4 to 6 stars at intermediate risk of bias, and those receiving less than 4 stars at high risk of bias. Quality and strength of evidence for each outcome were evaluated using the grading of recommendations, assessment, development, and evaluation (GRADE) approach.^15,16^ The GRADE profile was developed for outcomes that had 2 or more studies.

### Statistical Analysis

Data were combined across all studies according to pregnancy to estimate the pooled odds ratios (ORs) and the associated 95% CIs for the binary outcomes of live births and maternal adverse events. Unless otherwise specified, intervention groups included patients receiving the intervention of interest and no other treatment during the index pregnancy. If a treatment was discontinued prior to conception, the pregnancy was not included in that treatment group. Because the *JAK2* V617F mutation appears to have a causal role in thrombosis,^17^ a subgroup analysis was performed to examine whether the presence of the *JAK2* V617F mutation affected live births or adverse maternal complications. The analyses were based on a random effects model anticipating significant heterogeneity using the Mantel-Haenszel approach.^18^ Statistical heterogeneity was assessed using a χ^2^ test and quantified using the *I*^2^ statistic^19^; *I*^2^ values more than 50% and 75% were considered a priori to reflect moderate and high statistical heterogeneity, respectively. Findings were considered significant at 2-tailed *P* < .05. Data analyses were performed using Review Manager, version 5.3 (Cochrane Collaboration), and OpenMeta[Analyst].^20^

## Results

**Figure 1.**
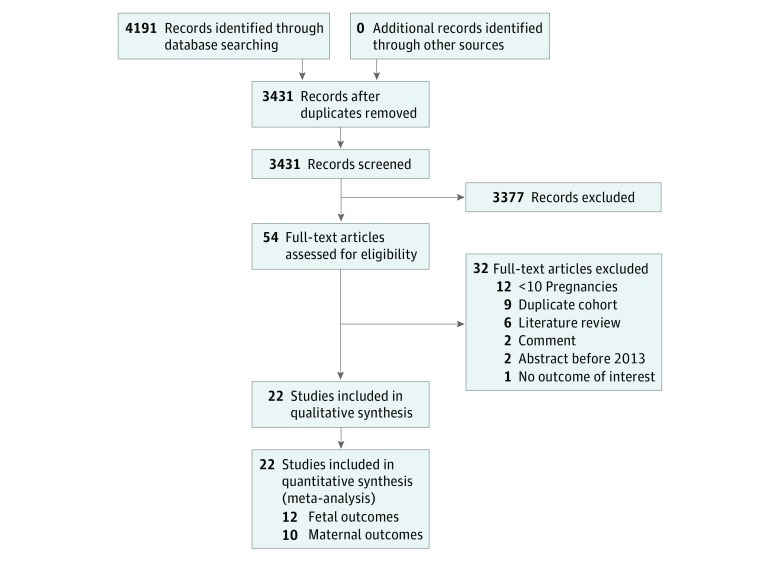
Study Selection

In total, 22 studies met the inclusion criteria. There were 12 studies that included comparisons of interventions and reported on fetal outcomes and 10 that included comparisons of interventions and reported on maternal outcomes. These investigations were included in the meta-analysis (Figure 1). The studies evaluated a total of 767 women and 1210 pregnancies. Fifteen of the studies included patients with essential thrombocythemia, 3 included patients with polycythemia vera, and 4 included patients with any classical MPN. There were no studies of patients exclusively with myelofibrosis that met our inclusion criteria. Most of the studies (19) were retrospective cohort design and 3 included a prospective cohort.^4,21,22^ None of the included studies were randomized clinical trials. Four studies were published as abstracts only^22,23,24,25^ and the remainder were published as peer-reviewed articles. Funding provided by peer-reviewed grants and industry was described in 2 studies^11,26^ and 1 study, respectively.^21^ The Table describes the characteristics of the studies.^3,4,8,9,10,11,21,22,23,24,25,26,27,28,29,30,31,32,33,34,35,36^

**Table.** Study Characteristics and Quality Based on the Newcastle-Ottawa Assessment Scale

Source	Single vs Multicenter	Diagnosis	Patients/Pregnancies, No.	Quality Assessment Based on the Newcastle-Ottawa Assessment Scale, No. of Stars Awarded				
				Selection	Comparability	Outcome	Total/8	Risk of Bias^a^
Alimam et al,^4^ 2016 United Kingdom	Multicenter	MPN	58/58	2	NA	3	5	Moderate
Bangerter et al,^8^ 2000 Germany	Single	ET	9/17	4	1	3	8	Low
Beauverd et al,^27^ 2016 United Kingdom	Multicenter	ET	8/10	2	NA	3	5	Moderate
Bertozzi et al,^28^ 2018 Italy	Multicenter	PV	15/25	2	NA	3	5	Moderate
Betti et al,^23^ 2015 Italy	Single	ET	38/62	1	NA	3	4	Moderate
Birgegård et al,^21^ 2018 Europe^e^	Multicenter	ET	40/54	2	NA	3	5	Moderate
Candoni et al,^29^ 2002 Italy	Single	ET	12/17	2	NA	3	5	Moderate
Cincotta et al,^9^ 2000 Australia	Multicenter	ET	12/30	2	NA	3	5	Moderate
Gangat et al,^10^ 2009 United States	Single	ET	36/63	3	NA	3	6	Moderate
Giona et al,^30^ 2012 Italy	Single	ET	6/15	3	NA	3	6	Moderate
Giraudet et al,^31^ 2011 France	Single	ET	13/18	3	NA	3	6	Moderate
Griesshammer et al,^24^ 2016 Europe	Multicenter	PV	48/121	2	NA	3	5	Moderate
Ianotto et al,^25^ 2018 United Kingdom	Single	MPN	13/39	2	NA	3	5	Moderate
Lapoirie et al^32^ 2018 France	Multicenter	MPN	14/27	3	NA	3	6	Moderate
Melillo et al,^11^ 2009 Italy	Multicenter	ET	92/122	3	NA	3	6	Moderate
Niittyvuopio et al,^33^ 2004 Finland	Multicenter	ET	16/40	1	NA	3	4	Moderate
Pagliaro et al,^34^ 1996 Italy	Single	ET	9/15	1	NA	3	4	Moderate
Passamonti et al,^35^ 2007 Italy	Multicenter	ET	62/103	2	NA	3	5	Moderate
Polushkina et al,^22^ 2014 Russia	Single	MPN	90/110	1	1	3	5	Moderate
Puyade et al,^36^ 2017 France	Single	ET	10/15	3	NA	3	6	Moderate
Randi et al,^26^ 2014 Italy	Multicenter	ET	158/237	3	NA	3	6	Moderate
Robinson et al,^3^ 2005 United Kingdom	Single	PV	8/25	4	NA	3	7	Low

Abbreviations: ET, essential thrombocythemia; MPN, myeloproliferative neoplasms; NA, not applicable; PV, polycythemia vera.

^a^ Scores 1 to 3 indicate high risk of bias; 4 to 6, moderate risk; 7 to 8, low risk.

Outcome data for evaluation of risk of bias were available for at least 90% of pregnancies. Two studies included comparable cohorts^8,22^ and 2 described the rationale for treatment.^3,8^ Two studies were considered to be at low risk of bias^3,8^ and all others were considered to be at moderate risk of bias. The risk of bias assessment according to the Newcastle-Ottawa Scale is summarized in the Table and support for the judgments for individual studies is available in eTable 3 in the Supplement.

Live births were reported in all studies and follow-up was at least to the end of the pregnancy. All 22 studies, including 1210 pregnancies, reported the number of live births. The overall live birth rate was 71.3% (95% CI, 65.1%-77.6%; among 1210 pregnancies). The live birth rate for essential thrombocythemia was 71.1% (95% CI, 65.6%-76.6%; among 815 pregnancies) and for polycythemia vera was 66.7% (95% CI, 59.4%-74.0%; among 159 pregnancies). In 10 studies reporting on 298 pregnancies, 59.1% (95% CI, 40.8%-77.3%) of spontaneous losses occurred in the first trimester, 24.9% (95% CI, 13.6%-36.1%) in the second trimester, and 12.3% (95% CI, 5.9%-18.8%) in the third trimester. Therapeutic abortions accounted for the remainder of lost pregnancies.

**Figure 2.**
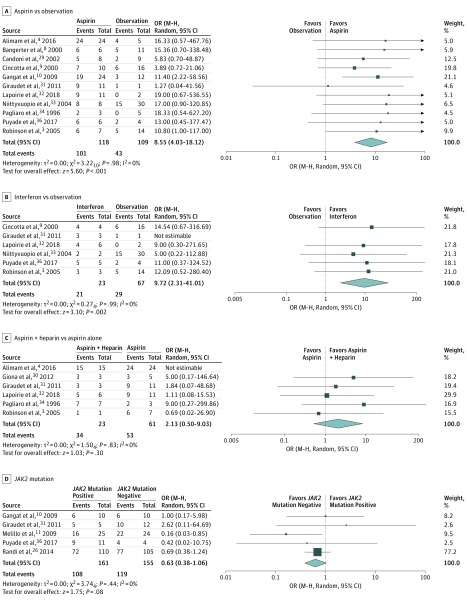
Live Births in Pregnant Patients With Myeloproliferative Neoplasms A, Aspirin vs observation. B, Interferon (with or without other interventions) vs observation. C, Aspirin with heparin vs aspirin alone. D, With and without the *JAK2* mutation. M-H indicates Mantel-Haenszel; OR, odds ratio. Size of box indicates the weight of the study on the meta-analysis result.

Aspirin use during pregnancy was associated with higher odds of live birth rate (11 studies, 227 patients; unadjusted OR, 8.6; 95% CI, 4.0-18.1; *I*^2^ = 0%) (Figure 2A). The aspirin dose was described in 7 studies and, in most cases, varied between individual patients. In 5 studies, patients received low-dose aspirin with a range of 50 to 160 mg and in 2 studies it was variable with individual patients receiving a range of doses from 75 to 300 mg. The addition of low doses of LMWH or unfractionated heparin to aspirin was not associated with significantly different odds of live birth (6 studies, 96 pregnancies; unadjusted OR, 2.1 for aspirin with heparin vs aspirin alone; 95% CI, 0.5-9.0; *I*^2^ = 0%) (Figure 2C). Only 1 study with 10 patients applicable to this analysis reported the use of unfractionated heparin^34^; the results were similar if this study was excluded and the analysis was restricted to LMWH (unadjusted OR, 1.6; 95% CI, 0.3-7.8; *I*^2^ = 0%). Administration of LMWH alone was not associated with significantly different odds of live birth (unadjusted OR, 6.0; 95% CI, 0.40-90.1; *I*^2^ = 0%) (eFigure 3 in the Supplement).

Interferon, with or without aspirin or heparin, increased the odds of live birth compared with observation alone (6 studies, 90 patients, unadjusted OR, 9.7; 95% CI, 2.3-41.0; *I*^2^ = 0%) (Figure 2B). The dose of interferon was not reported in these studies. Interferon alone, without concurrent aspirin and/or heparin, was only used in 1 study and 3 pregnancies, each of which resulted in live birth compared with 6 live births in the 16 pregnancies managed with observation alone.^9^ Four studies included patients who received interferon and aspirin (9 patients) vs aspirin alone (32 patients),^3,31,33,36^ and there was no difference in live births (unadjusted OR, 1.0; 95% CI, 0.1-11.6; *I*^2^ = 0%) (eFigure 6 in the Supplement). Three studies included patients receiving aspirin and heparin with (8 patients) or without (10 patients) interferon. In 2 of the studies, the live birth rate was the same in patients treated with and without interferon.^3,31^ In the third study, there were 3 live births in 5 pregnancies managed with the combination of interferon, aspirin, and LMWH and 5 live births in 6 pregnancies managed with aspirin and LMWH without interferon (eFigure 7 in the Supplement).^32^ Timing of treatment initiation was generally not reported. In 2 studies, the timing was described with initiation of treatment between 6 and 27 weeks’ gestation.^3,31^

The incidence of placental abruption was 1.2% (95% CI, 0.1%-2.5%; 6 studies, 329 pregnancies). None of the women were treated with interferon, 3 were managed with aspirin, 2 received only observation, and the treatment regimen was unclear in the remainder. There was no difference in the odds of the live birth rate in patients with a *JAK2* mutation compared with those with wild-type *JAK2* (OR, 0.6; 95% CI, 0.4-1.1; *I*^2^ = 0%; 5 studies, 316 patients) (Figure 2D)

Overall, 16 studies of 743 pregnancies reported data on the rate of any adverse events in pregnant women. The pooled rate of any adverse events was 9.6% (95% CI, 5.9%-13.3%). Ten of these studies included comparisons of interventions to allow for quantitative analysis. Maternal events did not differ among patients managed with aspirin or observation (10 studies, 198 pregnancies; unadjusted OR, 0.6; 95% CI, 0.2-1.8; *I*^2^ = 0%) (eFigure 8 in the Supplement) or aspirin combined with heparin vs observation (6 studies, 111 pregnancies; unadjusted OR, 1.1; 95% CI, 0.3-4.3; *I*^2^ = 0%) (eFigure 9 in the Supplement). Similarly, there was no difference in the odds of maternal events with the use of interferon (6 studies, 155 pregnancies; unadjusted OR, 1.5 for interferon vs no interferon; 95% CI, 0.5-4.5; *I*^2^ = 0%) (eFigure 14 in the Supplement). Four studies reported on the incidence of bleeding or thrombosis in patients treated with LMWH vs observation alone in the postpartum period, and there was no significant difference (4 studies, 95 pregnancies; unadjusted OR, 0.3; 95% CI, 0.04-2.6; *I*^2^ = 35%) (eFigure 18 in the Supplement). Additional analyses and information about other possible therapeutic combinations are available in eFigures 1, 2, 4, 5, 10-13, and 15-17 in the Supplement.

Preeclampsia was the most commonly reported adverse event (12 studies, 799 pregnancies) with a pooled incidence of 3.1% (95% CI, 1.7%-4.5%). Four studies reported the incidence of preeclampsia in pregnancies managed with aspirin compared with observation.^3,9,33,36^ In 64 pregnancies managed with observation alone, 7 were complicated by preeclampsia; in 31 pregnancies in which aspirin was used there were no reported cases of preeclampsia (4 studies, 95 pregnancies; OR, 0.4; 95% CI, 0.08-2.1). The pooled incidence of venous thrombosis was 1.5% (95% CI, 0.6%-2.4%) and the pooled incidence of arterial thrombosis was 1.3% (95% CI, 0.3%-2.3%). Use of aspirin was not associated with a difference in the odds of thrombosis in pregnancy (unadjusted OR, 0.8- 95% CI, 0.1%-4.3%). Only 1 study included patients treated with observation, heparin, or the combination of aspirin and heparin and the incidences of thrombotic events were 0 in 2 patients managed with observation, 0 in 1 patient who received heparin, and 1 in 7 patients treated with the combination of aspirin and heparin.^32^ Postpartum hemorrhage occurred in 1.5% (95% CI, 0.1%-2.9%) of pregnancies and other bleeding events occurred in 1.1% (0.2%-2.0%) of pregnancies.

The GRADE quality of evidence, relative effect, and anticipated absolute effects are summarized in eTable 4 in the Supplement. The quality of the evidence was moderate for the association of aspirin and interferon with live birth rate compared with observation alone owing to the large effect size. Quality was low to very low for other outcomes predominantly owing to risk of bias, imprecision, and inconsistency.

## Discussion

In this systematic review and meta-analysis, we identified a live birth rate of 71.3% in patients with MPNs. This rate is lower than the expected live birth rate of approximately 80% (excluding elective terminations) in the general population.^37^ Successful pregnancies occurred more frequently in patients with essential thrombocythemia (71.1%) than polycythemia vera (66.7%). Moderate-quality evidence suggests that the use of aspirin and interferon during pregnancy were associated with nearly 9- to 10-fold higher odds of a successful pregnancy than observation alone. Maternal adverse events were uncommon and use of aspirin or interferon was not associated with difference in the odds of maternal adverse outcomes.

Higher live birth rates associated with the use of aspirin and interferon suggest a potential reduction in disorders associated with placental dysfunction in MPN pregnancies. Placenta-mediated pregnancy complications are thought to arise early in pregnancy owing to inadequate trophoblast invasion of maternal spiral arteries creating a high-resistance, low-flow uteroplacental circulation.^38^ Preeclampsia has been reported to occur at higher frequencies in pregnant women with MPNs compared with other pregnant populations, in 5% to 11% in patients with essential thrombocythemia,^11,26^ and 12% to 17% in those with polycythemia vera.^3,32^ In our review, the overall incidence of preeclampsia was 3.1% (95% CI, 1.7%-4.5%), which is closer to the incidence of 1.45% to 4.0% in the general population,^39,40,41^ potentially because patients received treatment.

Aspirin has been advocated for prevention of uteroplacental insufficiency in high-risk groups because aspirin inhibits platelet aggregation and results in the release of nitric oxide and in a reduction of oxidative stress and inflammation.^38^ In a meta-analysis, aspirin appeared to reduce the risk of preterm birth in high-risk pregnancies (<37 weeks of gestation) by 14% (relative risk, 0.86; CI, 0.76-0.98), intrauterine growth restriction by 20% (RR, 0.80; CI, 0.65-0.99), and preeclampsia by 24% (relative risk, 0.76; 95% CI, 0.62-0.95).^42^ The increase in live birth rate in our review supports further studies addressing universal use of aspirin for all pregnancies with ET and PV.

The gestational age at which to commence aspirin use has been debated. Initiation at less than 16 weeks’ gestation has been associated with a reduction in pregnancies with fetal growth restriction and infants small for gestational age.^38^ A median of 12.7 weeks for initiation was reported by Rolnik and colleagues.^43^ Initiation of aspirin between 13 and 16 weeks’ gestation would not reduce the risk of first-trimester miscarriages that occur with MPNs; thus, if the intent is to prevent first-trimester loss, aspirin use should be considered early in pregnancy.

Aspirin use in pregnant patients with essential thrombocythemia was not associated with increases in bleeding events when used alone or with heparin. A review that included studies with 5 or more pregnancies with essential thrombocythemia reported similar results. In 191 pregnancies, bleeding occurred in 4.3% of women using aspirin (95% CI, 1.4%-8.7%) compared with 4.9% among 129 pregnancies without aspirin use (95% CI, 2.0%-9.0%).^44^ There were no antepartum bleeding events in 71 pregnancies using a combination of LMWH and aspirin (95% CI, 0.0%-6.2%).

The use of interferon is an accepted cytoreductive therapy during pregnancy for patients with MPNs.^5,7^ In a systematic review of 63 pregnancies (40 pregnancies in patients with essential thrombocythemia), interferon was not reported to be associated with fetal loss despite its use in 43 pregnancies in the first trimester and in 38 pregnancies during the entire antepartum interval.^45^ Interferon exhibits antiproliferative properties on a variety of cells and in essential thrombocythemia has been found to inhibit thrombopoietin-induced megakaryocyte growth.^46^ The antiproliferative effects may explain the higher live birth rate observed in the systematic review. Interferon has not generally been proposed as a first-line intervention in pregnant patients with essential thrombocythemia.^5,47^ The early addition of interferon to improve placental hypoperfusion may further improve pregnancy outcomes; however, further investigation with randomized clinical trials is needed to investigate this.

The live birth rate was not associated with combined use of heparin and aspirin in comparison with aspirin alone or with heparin compared with observation. Several algorithms suggest the use of heparin for pregnancy complications.^5,47^ However, the use of heparin for placenta-mediated pregnancy complications and recurrent miscarriages has been debated.^48,49,50^ Heparin has been theorized to reduce the risk of placenta-mediated complications because of its anti-inflammatory, antiangiogenic, and anticomplement effects.^51^ However, randomized clinical trials have not shown that heparin decreases recurrent miscarriages (except for patients with obstetric antiphospholipid antibody syndrome) or nonsevere, placenta-mediated pregnancy complications,^52^ but a benefit of heparin for the prevention of recurrent severe complications (early-onset preeclampsia, major placental abruption, ≤5th percentile growth or pregnancy loss at >20 weeks) has not yet been excluded.^50,51^

There was no association with *JAK2* mutation and live birth rate. To adhere to our prespecified definitions, studies that combined fetal losses with other pregnancy complications were excluded from the meta-analysis. This resulted in small numbers and the exclusion of some studies that have reported an adverse effect of the *JAK2* mutation on pregnancy outcomes (eg, Passamonti et al^35^). The *JAK2* mutation is a gain-of-function mutation associated with increased proliferation of hematopoietic stem cells. Whether the *JAK2* mutation warrants the use of interferon in all pregnancies could not be determined in this systematic review but merits further investigation. The calreticulin (*CALR*) gene is the second most common driver mutation in MPN and is associated with a lower risk of thrombosis than the *JAK2* mutation.^53,54^ Because most studies have reported on pregnancies before the 2013 discovery of the *CALR* mutation, whether pregnancy differs in *CALR*-mutated MPNs is unknown and needs further exploration.

### Limitations

This study has limitations. To our knowledge, this is the first systematic review of studies of interventions in pregnant women with MPNs. Limitations of this systematic review and meta-analysis are acknowledged, some owing to the data available for review. Most studies were retrospective and there is a possibility of ascertainment bias with early pregnancy losses being potentially underreported. Patients with essential thrombocythemia were predominantly represented compared with patients with polycythemia vera. The rationale for treatment allocation was generally not described or standardized and, although combined in the meta-analysis, may not have been indicated for all patients. Although interventions were not associated with improved or adverse maternal outcomes, maternal outcomes were infrequently reported. In nonpregnant patients, the use of aspirin to reduce thrombosis risk in essential thrombocythemia currently is of uncertain benefit and associated with an increased risk of bleeding.^55^ In pregnant women, aspirin to prevent thrombosis has been previously reported and was not associated with reduction of these events; 2.9% with aspirin use in 283 pregnancies (95% CI, 1.3%-5.1%) compared with 2.8% among 158 pregnancies without aspirin use (95% CI, 0.9%-5.8%).^44^ In addition, given that the studies span up to 4 decades, the outcomes may have been affected by changing standards of care.

## Conclusions

Moderate-quality evidence suggests that the use of aspirin alone or in combination with interferon in pregnancies affected by MPNs was associated with higher odds of live birth rate without significant maternal or fetal adverse effects. The use of heparin did not appear to be associated with higher odds of birth outcomes. Whether the presence of the *JAK2* mutation should be considered a risk that warrants interferon in addition to aspirin is yet to be determined. The prevalence of MPNs in pregnancy appears to be increasing; consequently, there may be an increased need to optimize management of these pregnancies. Efforts focused on establishing collaborations to risk-stratify pregnancies and assess the management of pregnancies systematically with prospective studies or registries are warranted.

## Notes

Supplement. **eTable 1.** Search Strategies **eTable 2.** Criteria for Risk of Bias Assessments **eTable 3.** Detailed Support for Risk of Bias Assessments **eFigure 1.** Live Births in Patients Managed With ASA + Heparin vs. Observation **eFigure 2.** Live Births in Patients Managed With ASA + Heparin vs. Heparin **eFigure 3.** Live Births in Patients Managed With Heparin vs. Observation **eFigure 4.** Live Births in Patients Managed With IFN vs. No IFN (With or Without Other Interventions) **eFigure 5.** Live Births in Patients Managed With IFN vs. Observation **eFigure 6.** Live Births in Patients Managed With IFN + ASA vs. ASA **eFigure 7.** Live Births in Patients Managed With IFN + ASA + Heparin vs. ASA + Heparin **eFigure 8.** Maternal Events in Patients Managed With ASA vs. Observation **eFigure 9.** Maternal Events in Patients Managed With ASA + Heparin vs. Observation **eFigure 10.** Maternal Events in Patients Managed With ASA + Heparin vs. ASA **eFigure 11.** Maternal Events in Patients Managed With ASA + Heparin vs. Heparin **eFigure 12.** Maternal Events in Patients Managed With Heparin vs. Observation **eFigure 13.** Maternal Events in Patients Managed With IFN (With or Without Other Interventions) vs. Observation **eFigure 14.** Maternal Events in Patients Managed With IFN vs. No IFN (With or Without Other Interventions) **eFigure 15.** Maternal Events in Patients Managed With IFN vs. Observation **eFigure 16.** Maternal Events in Patients Managed With IFN + ASA vs. ASA **eFigure 17.** Maternal Events in Patients Managed With IFN + ASA + Heparin vs. ASA + Heparin **eFigure 18.** Maternal Events in Patients Managed With Postpartum Heparin vs. No Postpartum Heparin **eTable 4.** Quality of Evidence for Live Birth Rates and Maternal Adverse Events
