# Supplementary material for: Association of Treatments for Myeloproliferative Neoplasms During Pregnancy With Birth Rates and Maternal Outcomes: A Systematic Review and Meta-analysis
Source: JAMA Netw Open. 2019 Oct 4;2(10):e1912666. doi: 10.1001/jamanetworkopen.2019.12666 (PMC6784750; doi:10.1001/jamanetworkopen.2019.12666)
Supplement: Supplement. — eTable 1. Search Strategies eTable 2. Criteria for Risk of Bias Assessments eTable 3. Detailed Support for Risk of Bias Assessments eFigure 1. Live Births in Patients Managed With ASA + Heparin vs. Observation eFigure 2. Live Births in Patients Managed With ASA + Heparin vs. Heparin eFigure 3. Live Births in Patients Managed With Heparin vs. Observation eFigure 4. Live Births in Patients Managed With IFN vs. No IFN (With or Without Other Interventions) eFigure 5. Live Births in Patients Managed With IFN vs. Observation eFigure 6. Live Births in Patients Managed With IFN + ASA vs. ASA eFigure 7. Live Births in Patients Managed With IFN + ASA + Heparin vs. ASA + Heparin eFigure 8. Maternal Events in Patients Managed With ASA vs. Observation eFigure 9. Maternal Events in Patients Managed With ASA + Heparin vs. Observation eFigure 10. Maternal Events in Patients Managed With ASA + Heparin vs. ASA eFigure 11. Maternal Events in Patients Managed With ASA + Heparin vs. Heparin eFigure 12. Maternal Events in Patients Managed With Heparin vs. Observation eFigure 13. Maternal Events in Patients Managed With IFN (With or Without Other Interventions) vs. Observation eFigure 14. Maternal Events in Patients Managed With IFN vs. No IFN (With or Without Other Interventions) eFigure 15. Maternal Events in Patients Managed With IFN vs. Observation eFigure 16. Maternal Events in Patients Managed With IFN + ASA vs. ASA eFigure 17. Maternal Events in Patients Managed With IFN + ASA + Heparin vs. ASA + Heparin eFigure 18. Maternal Events in Patients Managed With Postpartum Heparin vs. No Postpartum Heparin eTable 4. Quality of Evidence for Live Birth Rates and Maternal Adverse Events [file jamanetwopen-2-e1912666-s001.pdf]

Supplementary Online Content

Maze D, Kazi S, Gupta V, et al. Association of treatments for myeloproliferative neoplasms during pregnancy with birth rates and maternal outcomes: a systematic review and meta-analysis. *JAMA Netw Open*. 2019;2(10):e1912666. doi:10.1001/jamanetworkopen.2019.12666

eTable 1. Search Strategies

eTable 2. Criteria for Risk of Bias Assessments

eTable 3. Detailed Support for Risk of Bias Assessments

eFigure 1. Live Births in Patients Managed With ASA + Heparin vs. Observation

eFigure 2. Live Births in Patients Managed With ASA + Heparin vs. Heparin

eFigure 3. Live Births in Patients Managed With Heparin vs. Observation

eFigure 4. Live Births in Patients Managed With IFN vs. No IFN (With or Without Other Interventions)

eFigure 5. Live Births in Patients Managed With IFN vs. Observation

eFigure 6. Live Births in Patients Managed With IFN + ASA vs. ASA

eFigure 7. Live Births in Patients Managed With IFN + ASA + Heparin vs. ASA + Heparin

eFigure 8. Maternal Events in Patients Managed With ASA vs. Observation

eFigure 9. Maternal Events in Patients Managed With ASA + Heparin vs. Observation

eFigure 10. Maternal Events in Patients Managed With ASA + Heparin vs. ASA

eFigure 11. Maternal Events in Patients Managed With ASA + Heparin vs. Heparin

eFigure 12. Maternal Events in Patients Managed With Heparin vs. Observation

eFigure 13. Maternal Events in Patients Managed With IFN (With or Without Other Interventions) vs. Observation

eFigure 14. Maternal Events in Patients Managed With IFN vs. No IFN (With or Without Other Interventions)

eFigure 15. Maternal Events in Patients Managed With IFN vs. Observation

eFigure 16. Maternal Events in Patients Managed With IFN + ASA vs. ASA

eFigure 17. Maternal Events in Patients Managed With IFN + ASA + Heparin vs. ASA + Heparin

eFigure 18. Maternal Events in Patients Managed With Postpartum Heparin vs. No Postpartum Heparin

eTable 4. Quality of Evidence for Live Birth Rates and Maternal Adverse Events

This supplementary material has been provided by the authors to give readers additional information about their work.

© 2019 Maze D et al. *JAMA Network Open.*

eTable 1. Search strategies

Ovid MEDLINE(R) 1946 to September Week 2 2017

#	Searches	Results	Type
1	Myeloproliferative Disorders/	5730	Advanced
2	Anemia, Myelophthisic/	554	Advanced
3	Polycythemia Vera/	5986	Advanced
4	Primary Myelofibrosis/	5998	Advanced
5	Thrombocythemia, Essential/	2808	Advanced
6	myeloproliferat*.mp,kw.	11026	Advanced
7	myelo-proliferat*.mp,kw.	39	Advanced
8	myelophthis*.mp,kw.	595	Advanced
9	leu?oerythro*.mp,kw.	566	Advanced
10	leu?o-erythro*.mp,kw.	43	Advanced
11	(polycyth?em* adj2 vera?).mp,kw.	7495	Advanced
12	(polycyth?em* adj2 rubra?).mp,kw.	376	Advanced
13	(polycyth?em* adj2 cryptogen*).mp,kw.	0	Advanced
14	(polycyth?em* adj2 myelopath*).mp,kw.	0	Advanced
15	(polycyth?em* adj2 primar*).mp,kw.	235	Advanced
16	(polycyth?em* adj2 splenomegal*).mp,kw.	53	Advanced
17	erythr?em*.mp,kw.	548	Advanced
18	erythrocyt??em*.mp,kw.	66	Advanced
19	leleangiect*.mp,kw.	0	Advanced
20	osler-vaquez*.mp,kw.	3	Advanced
21	vaquez*.tw,kw.	150	Advanced
22	osler??.tw,kw.	2634	Advanced
23	myelofibros*.mp,kw.	7628	Advanced

© 2019 Maze D et al. *JAMA Network Open.*

24	(myeloid adj2 metaplas*).mp,kw.	1180	Advanced
25	myeloscleros*.mp,kw.	198	Advanced
26	(myelos* adj2 nonleuk?em*).mp,kw.	5	Advanced
27	(myelos* adj2 non-leuk?em*).mp,kw.	4	Advanced
28	(bone marrow? adj2 fibros*).mp,kw.	654	Advanced
29	(thrombocyt* adj2 essential*).mp,kw.	4382	Advanced
30	(thrombocyt* adj2 primar*).mp,kw.	931	Advanced
31	(thrombocyt* adj2 idiopath*).mp,kw.	8417	Advanced
32	(thrombocyt* adj3 dominan*).mp,kw.	51	Advanced
33	(thrombocyt* adj2 h?emorrhag*).mp,kw.	557	Advanced
34	hyperthrombocyt*.mp,kw.	28	Advanced
35	piastrinem*.mp,kw.	15	Advanced
36	or/1-35	35080	Advanced
37	Pregnancy/	837451	Advanced
38	exp Pregnancy Trimesters/	38975	Advanced
39	Pregnancy, High-Risk/	4744	Advanced
40	exp Pregnancy Complications/	405178	Advanced
41	Pregnancy Complications, Neoplastic/	7564	Advanced
42	exp Pregnancy Outcome/	51779	Advanced
43	Peripartum Period/	811	Advanced
44	exp Postpartum Period/	59230	Advanced
45	Prenatal Care/	24602	Advanced
46	exp Perinatal Care/	8680	Advanced
47	Postnatal Care/	4939	Advanced
48	Maternal Mortality/	9840	Advanced
49	Fetal Mortality/	469	Advanced
50	Maternal Death/	549	Advanced
51	exp Fetal Death/	29063	Advanced

52	pregnan*.mp,kw.	908151	Advanced
53	gestation*.mp,kw.	208393	Advanced
54	peripartum*.mp,kw.	3691	Advanced
55	peri-partum*.mp,kw.	147	Advanced
56	postpartum*.mp,kw.	56926	Advanced
57	post-partum*.mp,kw.	10367	Advanced
58	anten?tal*.mp,kw.	28979	Advanced
59	ante-n?tal*.mp,kw.	430	Advanced
60	pren?tal*.mp,kw.	152615	Advanced
61	pre-n?tal*.mp,kw.	1110	Advanced
62	perin?tal*.mp,kw.	63595	Advanced
63	peri-n?tal*.mp,kw.	192	Advanced
64	postn?tal*.mp,kw.	97272	Advanced
65	post-n?tal*.mp,kw.	6338	Advanced
66	abortion?.mp,kw.	85532	Advanced
67	miscarriag*.mp,kw.	10485	Advanced
68	(premature* adj2 birth?).mp,kw.	14308	Advanced
69	(pre-mature* adj2 birth?).mp,kw.	5	Advanced
70	live birth?.mp,kw.	19511	Advanced
71	stillbirth?.mp,kw.	10960	Advanced
72	(maternal* adj2 outcome?).mp,kw.	3570	Advanced
73	(fetal* adj2 outcome?).mp,kw.	4717	Advanced
74	(maternal* adj2 complicat*).mp,kw.	2795	Advanced
75	(fetal* adj2 complicat*).mp,kw.	2107	Advanced
76	(maternofetal* adj2 complicat*).mp,kw.	13	Advanced
77	(materno-fetal* adj2 complicat*).mp,kw.	16	Advanced
78	(maternofetal* adj2 outcome?).mp,kw.	13	Advanced
79	(materno-fetal* adj2 outcome?).mp,kw.	19	Advanced

© 2019 Maze D et al. *JAMA Network Open.*

80	(maternal* adj2 mortalit*).mp,kw.	14495	Advanced
81	(fetal* adj2 mortalit*).mp,kw.	2673	Advanced
82	(maternal* adj2 death?).mp,kw.	6155	Advanced
83	(fetal* adj2 death?).mp,kw.	28310	Advanced
84	(obstetric* adj3 complicat*).mp,kw.	20723	Advanced
85	(obstetric* adj3 outcome?).mp,kw.	3153	Advanced
86	or/37-85	1123328	Advanced
87	36 and 86	1289	Advanced
88	exp animals/ not (exp animals/ and exp humans/)	4584143	Advanced
89	87 not 88	1261	Advanced
90	remove duplicates from 89	1215	Advanced

© 2019 Maze D et al. *JAMA Network Open.*

Ovid MEDLINE(R) Epub Ahead of Print and In-Process & Other Non-Indexed Citations

#	Searches	Results	Type
1	myeloproliferat*.mp,kw.	1069	Advanced
2	myelo-proliferat*.mp,kw.	3	Advanced
3	myelophthis*.mp,kw.	7	Advanced
4	leu?oerythro*.mp,kw.	22	Advanced
5	leu?o-erythro*.mp,kw.	5	Advanced
6	(polycyth?em* adj2 vera?).mp,kw.	408	Advanced
7	(polycyth?em* adj2 rubra?).mp,kw.	31	Advanced
8	(polycyth?em* adj2 cryptogen*).mp,kw.	0	Advanced
9	(polycyth?em* adj2 myelopath*).mp,kw.	1	Advanced
10	(polycyth?em* adj2 primar*).mp,kw.	28	Advanced
11	(polycyth?em* adj2 splenomegal*).mp,kw.	9	Advanced
12	erythr?em*.mp,kw.	35	Advanced
13	erythrocyt??em*.mp,kw.	5	Advanced
14	leleangiect*.mp,kw.	0	Advanced
15	osler-vaquez*.mp,kw.	0	Advanced
16	vaquez*.tw,kw.	21	Advanced
17	osler??.tw,kw.	289	Advanced
18	myelofibros*.mp,kw.	613	Advanced
19	(myeloid adj2 metaplas*).mp,kw.	22	Advanced
20	myeloscleros*.mp,kw.	9	Advanced
21	(myelos* adj2 nonleuk?em*).mp,kw.	0	Advanced
22	(myelos* adj2 non-leuk?em*).mp,kw.	0	Advanced
23	(bone marrow? adj2 fibros*).mp,kw.	95	Advanced
24	(thrombocyt* adj2 essential*).mp,kw.	397	Advanced

© 2019 Maze D et al. *JAMA Network Open.*

25	(thrombocyt* adj2 primar*).mp,kw.	134	Advanced
26	(thrombocyt* adj2 idiopath*).mp,kw.	278	Advanced
27	(thrombocyt* adj3 dominan*).mp,kw.	1	Advanced
28	(thrombocyt* adj2 h?emorrhag*).mp,kw.	65	Advanced
29	hyperthrombocyt*.mp,kw.	1	Advanced
30	piastrinem*.mp,kw.	0	Advanced
31	or/1-30	2524	Advanced
32	pregnan*.mp,kw.	39124	Advanced
33	gestation*.mp,kw.	16005	Advanced
34	peripartum*.mp,kw.	574	Advanced
35	peri-partum*.mp,kw.	22	Advanced
36	postpartum*.mp,kw.	5060	Advanced
37	post-partum*.mp,kw.	1161	Advanced
38	anten?tal*.mp,kw.	3905	Advanced
39	ante-n?tal*.mp,kw.	114	Advanced
40	pren?tal*.mp,kw.	8109	Advanced
41	pre-n?tal*.mp,kw.	157	Advanced
42	perin?tal*.mp,kw.	6047	Advanced
43	peri-n?tal*.mp,kw.	22	Advanced
44	postn?tal*.mp,kw.	7518	Advanced
45	post-n?tal*.mp,kw.	764	Advanced
46	abortion?.mp,kw.	3513	Advanced
47	miscarriag*.mp,kw.	1316	Advanced
48	(premature* adj2 birth?).mp,kw.	470	Advanced
49	(pre-mature* adj2 birth?).mp,kw.	1	Advanced
50	live birth?.mp,kw.	2242	Advanced
51	stillbirth?.mp,kw.	1054	Advanced
52	(maternal* adj2 outcome?).mp,kw.	732	Advanced

© 2019 Maze D et al. *JAMA Network Open.*

53	(fetal* adj2 outcome?).mp,kw.	707	Advanced
54	(maternal* adj2 complicat*).mp,kw.	405	Advanced
55	(fetal* adj2 complicat*).mp,kw.	274	Advanced
56	(maternofetal* adj2 complicat*).mp,kw.	2	Advanced
57	(materno-fetal* adj2 complicat*).mp,kw.	3	Advanced
58	(maternofetal* adj2 outcome?).mp,kw.	1	Advanced
59	(materno-fetal* adj2 outcome?).mp,kw.	4	Advanced
60	(maternal* adj2 mortalit*).mp,kw.	1234	Advanced
61	(fetal* adj2 mortalit*).mp,kw.	163	Advanced
62	(maternal* adj2 death?).mp,kw.	720	Advanced
63	(fetal* adj2 death?).mp,kw.	447	Advanced
64	(obstetric* adj3 complicat*).mp,kw.	566	Advanced
65	(obstetric* adj3 outcome?).mp,kw.	525	Advanced
66	or/32-65	63721	Advanced
67	31 and 66	61	Advanced

© 2019 Maze D et al. *JAMA Network Open.*

Embase Classic+Embase 1947 to 2017 September 25

#	Searches	Results	Type
1	myeloproliferative disorder/	9832	Advanced
2	myeloproliferative neoplasm/	4298	Advanced
3	myelophthisic anemia/	556	Advanced
4	polycythemia vera/	10961	Advanced
5	myeloid metaplasia/	5066	Advanced
6	thrombocythemia/	6498	Advanced
7	myeloproliferat*.mp,kw.	20206	Advanced
8	myelo-proliferat*.mp,kw.	92	Advanced
9	myelophthis*.mp,kw.	684	Advanced
10	leu?oerythro*.mp,kw.	565	Advanced
11	leu?o-erythro*.mp,kw.	128	Advanced
12	(polycyth?em* adj2 vera?).mp,kw.	12128	Advanced
13	(polycyth?em* adj2 rubra?).mp,kw.	529	Advanced
14	(polycyth?em* adj2 cryptogen*).mp,kw.	1	Advanced
15	(polycyth?em* adj2 myelopath*).mp,kw.	0	Advanced
16	(polycyth?em* adj2 primar*).mp,kw.	336	Advanced
17	(polycyth?em* adj2 splenomegal*).mp,kw.	33	Advanced
18	erythr?em*.mp,kw.	751	Advanced
19	erythrocyt??em*.mp,kw.	116	Advanced
20	leleangiect*.mp,kw.	0	Advanced
21	osler-vaquez*.mp,kw.	3	Advanced
22	vaquez*.tw,kw.	265	Advanced
23	osler??.tw,kw.	3686	Advanced
24	myelofibros*.mp,kw.	12616	Advanced
25	(myeloid adj2 metaplas*).mp,kw.	5432	Advanced

© 2019 Maze D et al. *JAMA Network Open.*

26	myeloscleros*.mp,kw.	322	Advanced
27	(myelos* adj2 nonleuk?em*).mp,kw.	6	Advanced
28	(myelos* adj2 non-leuk?em*).mp,kw.	11	Advanced
29	(bone marrow? adj2 fibros*).mp,kw.	1151	Advanced
30	(thrombocyt* adj2 essential*).mp,kw.	6387	Advanced
31	(thrombocyt* adj2 primar*).mp,kw.	1555	Advanced
32	(thrombocyt* adj2 idiopath*).mp,kw.	15243	Advanced
33	(thrombocyt* adj3 dominan*).mp,kw.	85	Advanced
34	(thrombocyt* adj2 h?emorrhag*).mp,kw.	860	Advanced
35	hyperthrombocyt*.mp,kw.	62	Advanced
36	piastrinem*.mp,kw.	22	Advanced
37	or/1-36	58693	Advanced
38	exp pregnancy/	726701	Advanced
39	exp pregnancy disorder/	544021	Advanced
40	high risk pregnancy/	9827	Advanced
41	exp pregnancy complication/	127997	Advanced
42	pregnancy outcome/	46442	Advanced
43	perinatal period/	30224	Advanced
44	puerperium/	38218	Advanced
45	exp perinatal care/	52137	Advanced
46	exp postnatal care/	105201	Advanced
47	maternal mortality/	21809	Advanced
48	fetus mortality/	4181	Advanced
49	maternal death/	790	Advanced
50	exp fetus death/	40547	Advanced
51	pregnan*.mp,kw.	968751	Advanced
52	gestation*.mp,kw.	291027	Advanced

© 2019 Maze D et al. *JAMA Network Open.*

53	peripartum*.mp,kw.	5746	Advanced
54	peri-partum*.mp,kw.	314	Advanced
55	postpartum*.mp,kw.	63563	Advanced
56	post-partum*.mp,kw.	17588	Advanced
57	anten?tal*.mp,kw.	45219	Advanced
58	ante-n?tal*.mp,kw.	932	Advanced
59	pren?tal*.mp,kw.	235034	Advanced
60	pre-n?tal*.mp,kw.	1888	Advanced
61	perin?tal*.mp,kw.	127280	Advanced
62	peri-n?tal*.mp,kw.	375	Advanced
63	postn?tal*.mp,kw.	135567	Advanced
64	post-n?tal*.mp,kw.	10665	Advanced
65	abortion?.mp,kw.	120929	Advanced
66	miscarriag*.mp,kw.	19736	Advanced
67	(premature* adj2 birth?).mp,kw.	7685	Advanced
68	(pre-mature* adj2 birth?).mp,kw.	15	Advanced
69	live birth?.mp,kw.	31560	Advanced
70	stillbirth?.mp,kw.	20506	Advanced
71	(maternal* adj2 outcome?).mp,kw.	6277	Advanced
72	(fetal* adj2 outcome?).mp,kw.	7867	Advanced
73	(maternal* adj2 complicat*).mp,kw.	4734	Advanced
74	(fetal* adj2 complicat*).mp,kw.	3561	Advanced
75	(maternofetal* adj2 complicat*).mp,kw.	23	Advanced
76	(materno-fetal* adj2 complicat*).mp,kw.	24	Advanced
77	(maternofetal* adj2 outcome?).mp,kw.	24	Advanced
78	(materno-fetal* adj2 outcome?).mp,kw.	36	Advanced
79	(maternal* adj2 mortalit*).mp,kw.	25488	Advanced

© 2019 Maze D et al. *JAMA Network Open.*

80	(fetal* adj2 mortalit*).mp,kw.	2899	Advanced
81	(maternal* adj2 death?).mp,kw.	9456	Advanced
82	(fetal* adj2 death?).mp,kw.	10235	Advanced
83	(obstetric* adj3 complicat*).mp,kw.	7853	Advanced
84	(obstetric* adj3 outcome?).mp,kw.	5615	Advanced
85	or/38-84	1496723	Advanced
86	37 and 85	2542	Advanced
87	(exp animals/ or exp animal experimentation/ or nonhuman/) not ((exp animals/ or exp animal experimentation/ or nonhuman/) and exp human/)	6649052	Advanced
88	86 not 87	2475	Advanced
89	remove duplicates from 88	2413	Advanced

© 2019 Maze D et al. *JAMA Network Open.*

EBM Reviews - Cochrane Database of Systematic Reviews 2005 to September 20, 2017

#	Searches	Results	Type
1	myeloproliferat*.mp,kw.	21	Advanced
2	myelo-proliferat*.mp,kw.	0	Advanced
3	myelophthis*.mp,kw.	0	Advanced
4	leu?oerythro*.mp,kw.	1	Advanced
5	leu?o-erythro*.mp,kw.	0	Advanced
6	(polycyth?em* adj2 vera?).mp,kw.	9	Advanced
7	(polycyth?em* adj2 rubra?).mp,kw.	2	Advanced
8	(polycyth?em* adj2 cryptogen*).mp,kw.	0	Advanced
9	(polycyth?em* adj2 myelopath*).mp,kw.	0	Advanced
10	(polycyth?em* adj2 primar*).mp,kw.	0	Advanced
11	(polycyth?em* adj2 splenomegal*).mp,kw.	0	Advanced
12	erythr?em*.mp,kw.	1	Advanced
13	erythrocyt??em*.mp,kw.	0	Advanced
14	leleangiect*.mp,kw.	0	Advanced
15	osler-vaquez*.mp,kw.	2	Advanced
16	vaquez*.tw,kw.	2	Advanced
17	osler??.tw,kw.	14	Advanced
18	myelofibros*.mp,kw.	13	Advanced
19	(myeloid adj2 metaplas*).mp,kw.	3	Advanced
20	myeloscleros*.mp,kw.	3	Advanced
21	(myelos* adj2 nonleuk?em*).mp,kw.	2	Advanced
22	(myelos* adj2 non-leuk?em*).mp,kw.	0	Advanced
23	(bone marrow? adj2 fibros*).mp,kw.	4	Advanced
24	(thrombocyt* adj2 essential*).mp,kw.	8	Advanced
25	(thrombocyt* adj2 primar*).mp,kw.	3	Advanced

© 2019 Maze D et al. *JAMA Network Open.*

26	(thrombocyt* adj2 idiopath*).mp,kw.	21	Advanced
27	(thrombocyt* adj3 dominan*).mp,kw.	0	Advanced
28	(thrombocyt* adj2 h?emorrhag*).mp,kw.	43	Advanced
29	hyperthrombocyt*.mp,kw.	0	Advanced
30	piastrinem*.mp,kw.	0	Advanced
31	or/1-30	106	Advanced
32	pregnan*.mp,kw.	2227	Advanced
33	gestation*.mp,kw.	1197	Advanced
34	peripartum*.mp,kw.	41	Advanced
35	peri-partum*.mp,kw.	2	Advanced
36	postpartum*.mp,kw.	653	Advanced
37	post-partum*.mp,kw.	90	Advanced
38	anten?tal*.mp,kw.	522	Advanced
39	ante-n?tal*.mp,kw.	11	Advanced
40	pren?tal*.mp,kw.	300	Advanced
41	pre-n?tal*.mp,kw.	11	Advanced
42	perin?tal*.mp,kw.	878	Advanced
43	peri-n?tal*.mp,kw.	8	Advanced
44	postn?tal*.mp,kw.	592	Advanced
45	post-n?tal*.mp,kw.	58	Advanced
46	abortion?.mp,kw.	245	Advanced
47	miscarriag*.mp,kw.	324	Advanced
48	(premature* adj2 birth?).mp,kw.	270	Advanced
49	(pre-mature* adj2 birth?).mp,kw.	0	Advanced
50	live birth?.mp,kw.	340	Advanced
51	stillbirth?.mp,kw.	288	Advanced
52	(maternal* adj2 outcome?).mp,kw.	496	Advanced
53	(fetal* adj2 outcome?).mp,kw.	199	Advanced

© 2019 Maze D et al. *JAMA Network Open.*

54	(maternal* adj2 complicat*).mp,kw.	161	Advanced
55	(fetal* adj2 complicat*).mp,kw.	62	Advanced
56	(maternofetal* adj2 complicat*).mp,kw.	0	Advanced
57	(materno-fetal* adj2 complicat*).mp,kw.	0	Advanced
58	(maternofetal* adj2 outcome?).mp,kw.	0	Advanced
59	(materno-fetal* adj2 outcome?).mp,kw.	1	Advanced
60	(maternal* adj2 mortalit*).mp,kw.	295	Advanced
61	(fetal* adj2 mortalit*).mp,kw.	59	Advanced
62	(maternal* adj2 death?).mp,kw.	274	Advanced
63	(fetal* adj2 death?).mp,kw.	181	Advanced
64	(obstetric* adj3 complicat*).mp,kw.	100	Advanced
65	(obstetric* adj3 outcome?).mp,kw.	62	Advanced
66	or/32-65	2938	Advanced
67	31 and 66	30	Advanced

© 2019 Maze D et al. *JAMA Network Open.*

EBM Reviews - Cochrane Central Register of Controlled Trials August 2017

#	Searches	Results	Type
1	Myeloproliferative Disorders/	26	Advanced
2	Anemia, Myelophthisic/	0	Advanced
3	Polycythemia Vera/	48	Advanced
4	Primary Myelofibrosis/	39	Advanced
5	Thrombocythemia, Essential/	25	Advanced
6	myeloproliferat*.mp,kw.	223	Advanced
7	myelo-proliferat*.mp,kw.	1	Advanced
8	myelophthis*.mp,kw.	1	Advanced
9	leu?oerythro*.mp,kw.	0	Advanced
10	leu?o-erythro*.mp,kw.	0	Advanced
11	(polycyth?em* adj2 vera?).mp,kw.	200	Advanced
12	(polycyth?em* adj2 rubra?).mp,kw.	2	Advanced
13	(polycyth?em* adj2 cryptogen*).mp,kw.	0	Advanced
14	(polycyth?em* adj2 myelopath*).mp,kw.	0	Advanced
15	(polycyth?em* adj2 primar*).mp,kw.	13	Advanced
16	(polycyth?em* adj2 splenomegal*).mp,kw.	8	Advanced
17	erythr?em*.mp,kw.	3	Advanced
18	erythrocyt??em*.mp,kw.	6	Advanced
19	leleangiect*.mp,kw.	0	Advanced
20	osler-vaquez*.mp,kw.	0	Advanced
21	vaquez*.tw,kw.	0	Advanced
22	osler??.tw,kw.	57	Advanced
23	myelofibros*.mp,kw.	315	Advanced
24	(myeloid adj2 metaplas*).mp,kw.	75	Advanced
25	myeloscleros*.mp,kw.	0	Advanced

© 2019 Maze D et al. *JAMA Network Open.*

26	(myelos* adj2 nonleuk?em*).mp,kw.	0	Advanced
27	(myelos* adj2 non-leuk?em*).mp,kw.	0	Advanced
28	(bone marrow? adj2 fibros*).mp,kw.	32	Advanced
29	(thrombocyt* adj2 essential*).mp,kw.	154	Advanced
30	(thrombocyt* adj2 primar*).mp,kw.	88	Advanced
31	(thrombocyt* adj2 idiopath*).mp,kw.	410	Advanced
32	(thrombocyt* adj3 dominan*).mp,kw.	1	Advanced
33	(thrombocyt* adj2 h?emorrhag*).mp,kw.	151	Advanced
34	hyperthrombocyt*.mp,kw.	0	Advanced
35	piastrinem*.mp,kw.	0	Advanced
36	or/1-35	1200	Advanced
37	Pregnancy/	17845	Advanced
38	exp Pregnancy Trimesters/	1494	Advanced
39	Pregnancy, High-Risk/	143	Advanced
40	exp Pregnancy Complications/	7486	Advanced
41	Pregnancy Complications, Neoplastic/	12	Advanced
42	exp Pregnancy Outcome/	2661	Advanced
43	Peripartum Period/	7	Advanced
44	exp Postpartum Period/	1266	Advanced
45	Prenatal Care/	1092	Advanced
46	exp Perinatal Care/	438	Advanced
47	Postnatal Care/	321	Advanced
48	Maternal Mortality/	75	Advanced
49	Fetal Mortality/	2	Advanced
50	Maternal Death/	2	Advanced
51	exp Fetal Death/	184	Advanced
52	pregnan*.mp,kw.	32137	Advanced
53	gestation*.mp,kw.	13039	Advanced

© 2019 Maze D et al. *JAMA Network Open.*

54	peripartum*.mp,kw.	151	Advanced
55	peri-partum*.mp,kw.	7	Advanced
56	postpartum*.mp,kw.	4672	Advanced
57	post-partum*.mp,kw.	746	Advanced
58	anten?tal*.mp,kw.	2291	Advanced
59	ante-n?tal*.mp,kw.	25	Advanced
60	pren?tal*.mp,kw.	4041	Advanced
61	pre-n?tal*.mp,kw.	26	Advanced
62	perin?tal*.mp,kw.	3211	Advanced
63	peri-n?tal*.mp,kw.	4	Advanced
64	postn?tal*.mp,kw.	2565	Advanced
65	post-n?tal*.mp,kw.	160	Advanced
66	abortion?.mp,kw.	3359	Advanced
67	miscarriag*.mp,kw.	991	Advanced
68	(premature* adj2 birth?).mp,kw.	713	Advanced
69	(pre-mature* adj2 birth?).mp,kw.	0	Advanced
70	live birth?.mp,kw.	1338	Advanced
71	stillbirth?.mp,kw.	407	Advanced
72	(maternal* adj2 outcome?).mp,kw.	1354	Advanced
73	(fetal* adj2 outcome?).mp,kw.	516	Advanced
74	(maternal* adj2 complicat*).mp,kw.	467	Advanced
75	(fetal* adj2 complicat*).mp,kw.	184	Advanced
76	(maternofetal* adj2 complicat*).mp,kw.	0	Advanced
77	(materno-fetal* adj2 complicat*).mp,kw.	1	Advanced
78	(maternofetal* adj2 outcome?).mp,kw.	3	Advanced
79	(materno-fetal* adj2 outcome?).mp,kw.	0	Advanced
80	(maternal* adj2 mortalit*).mp,kw.	469	Advanced
81	(fetal* adj2 mortalit*).mp,kw.	70	Advanced

© 2019 Maze D et al. *JAMA Network Open.*

82	(maternal* adj2 death?).mp,kw.	238	Advanced
83	(fetal* adj2 death?).mp,kw.	364	Advanced
84	(obstetric* adj3 complicat*).mp,kw.	616	Advanced
85	(obstetric* adj3 outcome?).mp,kw.	504	Advanced
86	or/37-85	42216	Advanced
87	36 and 86	36	Advanced

© 2019 Maze D et al. *JAMA Network Open.*

eTable 2. Criteria for risk of bias assessments

Risk of Bias	Maximum stars	Support for Judgement
**Selection (1 star each, maximum 4 stars)**	**4 stars**	
Representativeness of the treated cohort	1 star	Star awarded if the study cohort was truly or somewhat representative of the MPN population in the community, e.g. if it was explicitly stated that all consecutive patients over a given time were included
Selection of the untreated cohort	1 star	Star awarded if the untreated cohort was drawn from the same community as the treated cohort and characteristics were likely similar
Ascertainment of intervention	1 star	Star awarded if data source was described, e.g. obstetrical records
Outcome not present at beginning of study	1 star	The outcome, live births, was not present at the beginning of the study by design and so all were awarded a star
**Comparability (1 star each, maximum 1 star)**	**1 star**	
Comparability of cohorts based on pregnancy risk	1 star	Star awarded if cohorts were balanced with respect to pregnancy risk {Griesshammer, 2018 #6}
**Outcome (1 star each, maximum 3 stars)**	**3 stars**	
Assessment of outcome	1 star	The outcome, live births, is unequivocal and so all were awarded a star
Was follow-up long enough?	1 star	Star awarded if data were available to the end of pregnancy
Adequacy of follow-up with data available for at least 90% of pregnancies	1 star	Star awarded if follow-up data were complete or available for at least 90% of pregnancies

eTable 3. Detailed support for risk of bias assessments

Alimam, 2016: Risk of bias based on the Newcastle-Ottawa Scale

Risk of Bias	Assessment	Support for Judgement
**Selection (1 star each, maximum 4 stars)**	**2 stars**	
Representativeness of the treated cohort	0 stars	Included only women who received care at UK hospitals with consultant-led maternity units
Selection of the untreated cohort	0 stars	The rationale for treatment was not described
Ascertainment of intervention	1 star	Data were collected prospectively using a data collection form
Outcome not present at beginning of study	1 star	The outcome, live births, was not present at the beginning of the study
**Comparability (1 star each, maximum 1 star)**	**0 stars**	
Comparability of cohorts based on pregnancy risk	0 stars	The characteristics of the cohorts were not described to enable the assessment of comparability
**Outcome (1 star each, maximum 3 stars)**	**3 stars**	
Assessment of outcome	1 star	The outcome, live births, is unequivocal; outcome data were collected prospectively using a data collection form
Was follow-up long enough?	1 star	Data were available to the end of pregnancy
Adequacy of follow-up with data available for at least 90% of pregnancies	1 star	Information was available for all pregnancies

© 2019 Maze D et al. *JAMA Network Open.*

Bangerter, 2000: Risk of bias based on the Newcastle-Ottawa Scale

Risk of Bias	Assessment	Support for Judgement
**Selection (1 star each, maximum 4 stars)**	**4 stars**	
Representativeness of the treated cohort	1 star	All patients with ET pregnancies during the study period were included; all patients with pregnancies beginning in 1995 were treated
Selection of the untreated cohort	1 star	All patients with pregnancies after 1995 were treated with ASA; increasing platelet count was the rationale for cytoreductive therapy in 3 patients
Ascertainment of intervention	1 star	Obstetric history, including management, was available for all patients
Outcome not present at beginning of study	1 star	The outcome, live births, was not present at the beginning of the study
**Comparability (1 star each, maximum 1 star)**	**0 stars**	
Comparability of cohorts based on pregnancy risk	0 stars	The characteristics of the cohorts were not described to enable the assessment of comparability
**Outcome (1 star each, maximum 3 stars)**	**3 stars**	
Assessment of outcome	1 star	The outcome, live births, is unequivocal
Was follow-up long enough?	1 star	Data were available to the end of pregnancy
Adequacy of follow-up with data available for at least 90% of pregnancies	1 star	Information was available for all pregnancies

ET, essential thrombocythemia; ASA, aspirin

© 2019 Maze D et al. *JAMA Network Open.*

Beauverd, 2016: Risk of bias based on the Newcastle-Ottawa Scale

Risk of Bias	Assessment	Support for Judgement
**Selection (1 star each, maximum 4 stars)**	**2 stars**	
Representativeness of the treated cohort	0 stars	Reported on ET pregnancies treated with IFN with pregnancy information available
Selection of the untreated cohort	0 stars	No untreated cohort
Ascertainment of intervention	1 star	Data extracted from patient records
Outcome not present at beginning of study	1 star	The outcome, live births, was not present at the beginning of the study
**Comparability (1 star each, maximum 1 star)**	**0 stars**	
Comparability of cohorts based on pregnancy risk	0 stars	There was only 1 (treated) cohort
**Outcome (1 star each, maximum 3 stars)**	**3 stars**	
Assessment of outcome	1 star	The outcome, live births, is unequivocal
Was follow-up long enough?	1 star	Data were available to the end of pregnancy
Adequacy of follow-up with data available for at least 90% of pregnancies	1 star	Information was available for all pregnancies

ET, essential thrombocythemia; IFN, interferon

© 2019 Maze D et al. *JAMA Network Open.*

Betti, 2015: Risk of bias based on the Newcastle-Ottawa Scale

Risk of Bias	Assessment	Support for Judgement
**Selection (1 star each, maximum 4 stars)**	**1 star**	
Representativeness of the treated cohort	0 stars	It is not clear how the cohort was selected
Selection of the untreated cohort	0 stars	The rationale for treatment was not described
Ascertainment of intervention	0 stars	Not described
Outcome not present at beginning of study	1 star	The outcome, live births, was not present at the beginning of the study
**Comparability (1 star each, maximum 1 star)**	**0 stars**	
Comparability of cohorts based on pregnancy risk	0 stars	The characteristics of the cohorts were not described to enable the assessment of comparability
**Outcome (1 star each, maximum 3 stars)**	**3 stars**	
Assessment of outcome	1 star	The outcome, live births, is unequivocal
Was follow-up long enough?	1 star	Data were available to the end of pregnancy
Adequacy of follow-up with data available for at least 90% of pregnancies	1 star	Information was available for 59/62 (95%) pregnancies

© 2019 Maze D et al. *JAMA Network Open.*

Bertozzi, 2018: Risk of bias based on the Newcastle-Ottawa Scale

Risk of Bias	Assessment	Support for Judgement
**Selection (1 star each, maximum 4 stars)**	**2 stars**	
Representativeness of the treated cohort	0 stars	It is not clear how the cohort was selected
Selection of the untreated cohort	0 stars	There was no explanation as to why some patients were untreated
Ascertainment of intervention	1 star	A complete medical history was obtained for each patient
Outcome not present at beginning of study	1 star	The outcome, live births, was not present at the beginning of the study
**Comparability (1 star each, maximum 1 star)**	**0 stars**	
Comparability of cohorts based on pregnancy risk	0 stars	The characteristics of the cohorts were not described to enable the assessment of comparability
**Outcome (1 star each, maximum 3 stars)**	**3 stars**	
Assessment of outcome	1 star	The outcome, live births, is unequivocal
Was follow-up long enough?	1 star	Data were available to the end of pregnancy
Adequacy of follow-up with data available for at least 90% of pregnancies	1 star	Information was available for all pregnancies

© 2019 Maze D et al. *JAMA Network Open.*

Birgegard, 2018: Risk of bias based on the Newcastle-Ottawa Scale

Risk of Bias	Assessment	Support for Judgement
**Selection (1 star each, maximum 4 stars)**	**2 stars**	
Representativeness of the treated cohort	0 stars	Only high risk patients (platelets > 1000 x 10^9/L and/or previous thrombohemorrhagic event) were included in the EXELS study
Selection of the untreated cohort	0 stars	The rationale for treatment was not described
Ascertainment of intervention	1 star	Data were collected prospectively at baseline and every 6 months for 5 years
Outcome not present at beginning of study	1 star	The outcome, live births, was not present at the beginning of the study
**Comparability (1 star each, maximum 1 star)**	**0 stars**	
Comparability of cohorts based on pregnancy risk	0 stars	The characteristics of the cohorts were not described to enable the assessment of comparability
**Outcome (1 star each, maximum 3 stars)**	**3 stars**	
Assessment of outcome	1 star	The outcome, live births, is unequivocal
Was follow-up long enough?	1 star	Data were available to the end of pregnancy
Adequacy of follow-up with data available for at least 90% of pregnancies	1 star	Information was available for 52/54 (96%) pregnancies

EXELS, Evaluation of Anagrelide Efficacy and Long-Term Safety

© 2019 Maze D et al. *JAMA Network Open.*

Candoni, 2002: Risk of bias based on the Newcastle-Ottawa Scale

Risk of Bias	Assessment	Support for Judgement
**Selection (1 star each, maximum 4 stars)**	**2 stars**	
Representativeness of the treated cohort	1 star	All patients with ET pregnancies during the study period were included
Selection of the untreated cohort	0 stars	The rationale for treatment was not described
Ascertainment of intervention	0 stars	Not described
Outcome not present at beginning of study	1 star	The outcome, live births, was not present at the beginning of the study
**Comparability (1 star each, maximum 1 star)**	**0 stars**	
Comparability of cohorts based on pregnancy risk	0 stars	The characteristics of the cohorts were not described to enable the assessment of comparability
**Outcome (1 star each, maximum 3 stars)**	**3 stars**	
Assessment of outcome	1 star	The outcome, live births, is unequivocal
Was follow-up long enough?	1 star	Data were available to the end of pregnancy
Adequacy of follow-up with data available for at least 90% of pregnancies	1 star	Information was available for all pregnancies

ET, essential thrombocythemia

© 2019 Maze D et al. *JAMA Network Open.*

Cincotta, 2000: Risk of bias based on the Newcastle-Ottawa Scale

Risk of Bias	Assessment	Support for Judgement
**Selection (1 star each, maximum 4 stars)**	**2 stars**	
Representativeness of the treated cohort	1 star	All patients with ET pregnancies during the study period were included
Selection of the untreated cohort	0 stars	The rationale for treatment was not described
Ascertainment of intervention	0 stars	Not described
Outcome not present at beginning of study	1 star	The outcome, live births, was not present at the beginning of the study
**Comparability (1 star each, maximum 1 star)**	**0 stars**	
Comparability of cohorts based on pregnancy risk	0 stars	The characteristics of the cohorts were not described to enable the assessment of comparability
**Outcome (1 star each, maximum 3 stars)**	**3 stars**	
Assessment of outcome	1 star	The outcome, live births, is unequivocal
Was follow-up long enough?	1 star	Data were available to the end of pregnancy
Adequacy of follow-up with data available for at least 90% of pregnancies	1 star	Information was available for all pregnancies

ET, essential thrombocythemia

© 2019 Maze D et al. *JAMA Network Open.*

Gangat, 2009: Risk of bias based on the Newcastle-Ottawa Scale

Risk of Bias	Assessment	Support for Judgement
**Selection (1 star each, maximum 4 stars)**	**3 stars**	
Representativeness of the treated cohort	1 star	All patients with ET pregnancies during the study period were included
Selection of the untreated cohort	0 stars	The rationale for treatment was not described
Ascertainment of intervention	1 star	Data were extracted from medical records
Outcome not present at beginning of study	1 star	The outcome, live births, was not present at the beginning of the study
**Comparability (1 star each, maximum 1 star)**	**0 stars**	
Comparability of cohorts based on pregnancy risk	0 stars	The characteristics of the cohorts were not described to enable the assessment of comparability
**Outcome (1 star each, maximum 3 stars)**	**3 stars**	
Assessment of outcome	1 star	The outcome, live births, is unequivocal
Was follow-up long enough?	1 star	Data were available to the end of pregnancy
Adequacy of follow-up with data available for at least 90% of pregnancies	1 star	Information was available for all pregnancies

ET, essential thrombocythemia

© 2019 Maze D et al. *JAMA Network Open.*

Giona, 2012: Risk of bias based on the Newcastle-Ottawa Scale

Risk of Bias	Assessment	Support for Judgement
**Selection (1 star each, maximum 4 stars)**	**3 stars**	
Representativeness of the treated cohort	1 star	All patients with ET pregnancies during the study period were included
Selection of the untreated cohort	0 stars	The rationale for treatment was not described
Ascertainment of intervention	1 star	Medical records were reviewed
Outcome not present at beginning of study	1 star	The outcome, live births, was not present at the beginning of the study
**Comparability (1 star each, maximum 1 star)**	**0 stars**	
Comparability of cohorts based on pregnancy risk	0 stars	The characteristics of the cohorts were not described to enable the assessment of comparability
**Outcome (1 star each, maximum 3 stars)**	**3 stars**	
Assessment of outcome	1 star	The outcome, live births, is unequivocal
Was follow-up long enough?	1 star	Data were available to the end of pregnancy
Adequacy of follow-up with data available for at least 90% of pregnancies	1 star	Information was available for all pregnancies

ET, essential thrombocythemia

© 2019 Maze D et al. *JAMA Network Open.*

Giraudet, 2011: Risk of bias based on the Newcastle-Ottawa Scale

Risk of Bias	Assessment	Support for Judgement
**Selection (1 star each, maximum 4 stars)**	**3 stars**	
Representativeness of the treated cohort	1 star	All ET pregnancies during the study period were included
Selection of the untreated cohort	0 stars	There was no untreated cohort
Ascertainment of intervention	1 star	Medical records were reviewed
Outcome not present at beginning of study	1 star	The outcome, live births, was not present at the beginning of the study
**Comparability (1 star each, maximum 1 star)**	**0 stars**	
Comparability of cohorts based on pregnancy risk	0 stars	The characteristics of the cohorts were not described to enable the assessment of comparability
**Outcome (1 star each, maximum 3 stars)**	**3 stars**	
Assessment of outcome	1 star	The outcome, live births, is unequivocal
Was follow-up long enough?	1 star	Data were available to the end of pregnancy
Adequacy of follow-up with data available for at least 90% of pregnancies	1 star	Information was available for all pregnancies

ET, essential thrombocythemia

© 2019 Maze D et al. *JAMA Network Open.*

Greisshammer, 2016: Risk of bias based on the Newcastle-Ottawa Scale

Risk of Bias	Assessment	Support for Judgement
**Selection (1 star each, maximum 4 stars)**	**2 stars**	
Representativeness of the treated cohort	1 star	All PV pregnancies reported to the European Leukemia Net during the study period were included
Selection of the untreated cohort	0 stars	It is unclear if the group with pregnancies prior to PV diagnosis had similar characteristics to those diagnosed pre-pregnancy
Ascertainment of intervention	0 stars	Not described
Outcome not present at beginning of study	1 star	The outcome, live births, was not present at the beginning of the study
**Comparability (1 star each, maximum 1 star)**	**0 stars**	
Comparability of cohorts based on pregnancy risk	0 star	The characteristics of the cohorts were not described to enable the assessment of comparability
**Outcome (1 star each, maximum 3 stars)**	**3 stars**	
Assessment of outcome	1 star	The outcome, live births, is unequivocal
Was follow-up long enough?	1 star	Data were available to the end of pregnancy
Adequacy of follow-up with data available for at least 90% of pregnancies	1 star	Information was available for all pregnancies

PV, polycythemia vera

© 2019 Maze D et al. *JAMA Network Open.*

Ianotto, 2018: Risk of bias based on the Newcastle-Ottawa Scale

Risk of Bias	Assessment	Support for Judgement
**Selection (1 star each, maximum 4 stars)**	**2 stars**	
Representativeness of the treated cohort	1 star	All ET pregnancies during the study period were included
Selection of the untreated cohort	0 stars	The rationale for treatment was not described
Ascertainment of intervention	0 stars	Not described
Outcome not present at beginning of study	1 star	The outcome, live births, was not present at the beginning of the study
**Comparability (1 star each, maximum 1 star)**	**0 stars**	
Comparability of cohorts based on pregnancy risk	0 stars	The characteristics of the cohorts were not described to enable the assessment of comparability
**Outcome (1 star each, maximum 3 stars)**	**3 stars**	
Assessment of outcome	1 star	The outcome, live births, is unequivocal
Was follow-up long enough?	1 star	Data were available to the end of pregnancy
Adequacy of follow-up with data available for at least 90% of pregnancies	1 star	Information was available for 92% of pregnancies

ET, essential thrombocythemia

© 2019 Maze D et al. *JAMA Network Open.*

Lapoirie, 2018: Risk of bias based on the Newcastle-Ottawa Scale

Risk of Bias	Assessment	Support for Judgement
**Selection (1 star each, maximum 4 stars)**	**3 stars**	
Representativeness of the treated cohort	1 star	All patients with MPN pregnancies during the study period were included
Selection of the untreated cohort	0 stars	The rationale for treatment was not described
Ascertainment of intervention	1 star	Data extracted from files
Outcome not present at beginning of study	1 star	The outcome, live births, was not present at the beginning of the study
**Comparability (1 star each, maximum 1 star)**	**0 stars**	
Comparability of cohorts based on pregnancy risk	0 stars	The characteristics of the cohorts were not described to enable the assessment of comparability
**Outcome (1 star each, maximum 3 stars)**	**3 stars**	
Assessment of outcome	1 star	The outcome, live births, is unequivocal
Was follow-up long enough?	1 star	Data were available to the end of pregnancy
Adequacy of follow-up with data available for at least 90% of pregnancies	1 star	Information was available for all pregnancies

MPN, myeloproliferative neoplasm

© 2019 Maze D et al. *JAMA Network Open.*

Melillo, 2018: Risk of bias based on the Newcastle-Ottawa Scale

Risk of Bias	Assessment	Support for Judgement
**Selection (1 star each, maximum 4 stars)**	**3 stars**	
Representativeness of the treated cohort	1 star	All patients with MPN pregnancies during the study period were included
Selection of the untreated cohort	0 stars	The rationale for treated was described but not standardized
Ascertainment of intervention	1 star	Data extracted from registry
Outcome not present at beginning of study	1 star	The outcome, live births, was not present at the beginning of the study
**Comparability (1 star each, maximum 1 star)**	**0 stars**	
Comparability of cohorts based on pregnancy risk	0 stars	The rationale for the use of IFN was described for those using IFN but the rationale for use of other interventions was not described
**Outcome (1 star each, maximum 3 stars)**	**3 stars**	
Assessment of outcome	1 star	The outcome, live births, is unequivocal
Was follow-up long enough?	1 star	Data were available to the end of pregnancy
Adequacy of follow-up with data available for at least 90% of pregnancies	1 star	Information was available for all pregnancies

MPN, myeloproliferative neoplasm; IFN, interferon

© 2019 Maze D et al. *JAMA Network Open.*

Nittyvuopio, 2004: Risk of bias based on the Newcastle-Ottawa Scale

Risk of Bias	Assessment	Support for Judgement
**Selection (1 star each, maximum 4 stars)**	**1 star**	
Representativeness of the treated cohort	0 stars	It is not clear how the cohort was selected
Selection of the untreated cohort	0 stars	The rationale for treatment was not described
Ascertainment of intervention	0 stars	Not described
Outcome not present at beginning of study	1 star	The outcome, live births, was not present at the beginning of the study
**Comparability (1 star each, maximum 1 star)**	**0 stars**	
Comparability of cohorts based on pregnancy risk	0 stars	The characteristics of the cohorts were not described to enable the assessment of comparability
**Outcome (1 star each, maximum 3 stars)**	**3 stars**	
Assessment of outcome	1 star	The outcome, live births, is unequivocal
Was follow-up long enough?	1 star	Data were available to the end of pregnancy
Adequacy of follow-up with data available for at least 90% of pregnancies	1 star	Information was available for all pregnancies

© 2019 Maze D et al. *JAMA Network Open.*

Pagliaro, 1996: Risk of bias based on the Newcastle-Ottawa Scale

Risk of Bias	Assessment	Support for Judgement
**Selection (1 star each, maximum 4 stars)**	**1 star**	
Representativeness of the treated cohort	0 stars	It is not clear how the cohort was selected
Selection of the untreated cohort	0 stars	The rationale for treatment was not described
Ascertainment of intervention	0 stars	Not described
Outcome not present at beginning of study	1 star	The outcome, live births, was not present at the beginning of the study
**Comparability (1 star each, maximum 1 star)**	**0 stars**	
Comparability of cohorts based on pregnancy risk	0 stars	The characteristics of the cohorts were not described to enable the assessment of comparability
**Outcome (1 star each, maximum 3 stars)**	**3 stars**	
Assessment of outcome	1 star	The outcome, live births, is unequivocal
Was follow-up long enough?	1 star	Data were available to the end of pregnancy
Adequacy of follow-up with data available for at least 90% of pregnancies	1 star	Information was available for all pregnancies

© 2019 Maze D et al. *JAMA Network Open.*

Passamonti, 2007: Risk of bias based on the Newcastle-Ottawa Scale

Risk of Bias	Assessment	Support for Judgement
**Selection (1 star each, maximum 4 stars)**	**2 stars**	
Representativeness of the treated cohort	1 star	Consecutive patients with ET pregnancies during the study period were included
Selection of the untreated cohort	0 stars	The rationale for treatment was not described
Ascertainment of intervention	0 stars	Not described
Outcome not present at beginning of study	1 star	The outcome, live births, was not present at the beginning of the study
**Comparability (1 star each, maximum 1 star)**	**0 stars**	
Comparability of cohorts based on pregnancy risk	0 stars	The characteristics of the cohorts were not described to enable the assessment of comparability
**Outcome (1 star each, maximum 3 stars)**	**3 stars**	
Assessment of outcome	1 star	The outcome, live births, is unequivocal
Was follow-up long enough?	1 star	Data were available to the end of pregnancy
Adequacy of follow-up with data available for at least 90% of pregnancies	1 star	Information was available for all pregnancies

ET, essential thrombocythemia

© 2019 Maze D et al. *JAMA Network Open.*

Polushkina, 2014: Risk of bias based on the Newcastle-Ottawa Scale

Risk of Bias	Assessment	Support for Judgement
**Selection (1 star each, maximum 4 stars)**	**1 star**	
Representativeness of the treated cohort	0 stars	It is not clear how the cohort was selected
Selection of the untreated cohort	0 stars	It is unclear if the retrospective group was similar to the prospective group
Ascertainment of intervention	0 stars	Not described
Outcome not present at beginning of study	1 star	The outcome, live births, was not present at the beginning of the study
**Comparability (1 star each, maximum 1 star)**	**1 star**	
Comparability of cohorts based on pregnancy risk	1 star	Cohorts defined by the development of the protocol and not pregnancy risk
**Outcome (1 star each, maximum 3 stars)**	**3 stars**	
Assessment of outcome	1 star	The outcome, live births, is unequivocal
Was follow-up long enough?	1 star	Data were available to the end of pregnancy
Adequacy of follow-up with data available for at least 90% of pregnancies	1 star	Information was available for all pregnancies

© 2019 Maze D et al. *JAMA Network Open.*

Puyade, 2017: Risk of bias based on the Newcastle-Ottawa Scale

Risk of Bias	Assessment	Support for Judgement
**Selection (1 star each, maximum 4 stars)**	**3 stars**	
Representativeness of the treated cohort	1 star	All patients with MPN pregnancies during the study period were included
Selection of the untreated cohort	0 stars	The rationale for treatment was not described
Ascertainment of intervention	1 star	Data were extracted from records
Outcome not present at beginning of study	1 star	The outcome, live births, was not present at the beginning of the study
**Comparability (1 star each, maximum 1 star)**	**0 stars**	
Comparability of cohorts based on pregnancy risk	0 stars	The rationale for the use of IFN was described but there were no patients with similar characteristics not treated with IFN
**Outcome (1 star each, maximum 3 stars)**	**3 stars**	
Assessment of outcome	1 star	The outcome, live births, is unequivocal
Was follow-up long enough?	1 star	Data were available to the end of pregnancy
Adequacy of follow-up with data available for at least 90% of pregnancies	1 star	Information was available for all pregnancies

MPN, myeloproliferative neoplasm; IFN, interferon

© 2019 Maze D et al. *JAMA Network Open.*

Randi, 2014: Risk of bias based on the Newcastle-Ottawa Scale

Risk of Bias	Assessment	Support for Judgement
**Selection (1 star each, maximum 4 stars)**	**3 stars**	
Representativeness of the treated cohort	1 star	The cohort was selected from the GIMEMA cohort study that included all patients with ET from 1980 - 2009
Selection of the untreated cohort	0 stars	The rationale for treatment was not described
Ascertainment of intervention	1 star	The centres had to complete patient and laboratory data on pre-specified spreadsheets
Outcome not present at beginning of study	1 star	The outcome, live births, was not present at the beginning of the study
**Comparability (1 star each, maximum 1 star)**	**0 stars**	
Comparability of cohorts based on pregnancy risk	0 stars	The characteristics of the cohorts were not described to enable the assessment of comparability
**Outcome (1 star each, maximum 3 stars)**	**3 stars**	
Assessment of outcome	1 star	The outcome, live births, is unequivocal
Was follow-up long enough?	1 star	Data were available to the end of pregnancy
Adequacy of follow-up with data available for at least 90% of pregnancies	1 star	Information was available for all pregnancies

GIMEMA, Gruppo Italiano Malattie EMatologiche dell'Adulto; ET, essential thrombocythemia

© 2019 Maze D et al. *JAMA Network Open.*

Robinson, 2005: Risk of bias based on the Newcastle-Ottawa Scale

Risk of Bias	Assessment	Support for Judgement
**Selection (1 star each, maximum 4 stars)**	**4 stars**	
Representativeness of the treated cohort	1 star	The cohort was selected from a specialty clinic
Selection of the untreated cohort	1 star	The rationale for treatment was described
Ascertainment of intervention	1 star	Data were extracted from medical records
Outcome not present at beginning of study	1 star	The outcome, live births, was not present at the beginning of the study
**Comparability (1 star each, maximum 1 star)**	**0 stars**	
Comparability of cohorts based on pregnancy risk	0 stars	The characteristics of the cohorts were not described to enable the assessment of comparability
**Outcome (1 star each, maximum 3 stars)**	**3 stars**	
Assessment of outcome	1 star	The outcome, live births, is unequivocal
Was follow-up long enough?	1 star	Data were available to the end of pregnancy
Adequacy of follow-up with data available for at least 90% of pregnancies	1 star	Information was available for all pregnancies

© 2019 Maze D et al. *JAMA Network Open.*

Study or Subgroup	ASA+Heparin Events	ASA+Heparin Total	Observation Events	Observation Total	Weight	Odds Ratio M–H, Random, 95% CI
Alimam 2016	15	15	4	5	27.8%	10.33 [0.36, 299.97]
Giraudet 2011	3	3	1	1		Not estimable
Lapoirie 2018	5	6	0	2	25.3%	18.33 [0.54, 627.20]
Paglario 1996	7	7	0	5	19.0%	165.00 [2.81, 9675.65]
Robinson 2005	1	1	5	14	27.8%	5.18 [0.18, 150.45]
**Total (95% CI)**		**32**		**27**	**100.0%**	**16.71 [2.83, 98.78]**
Total events	31		10			

Heterogeneity: Tau² = 0.00; Chi² = 1.76, df = 3 (P = 0.62); I² = 0%
Test for overall effect: Z = 3.11 (P = 0.002)

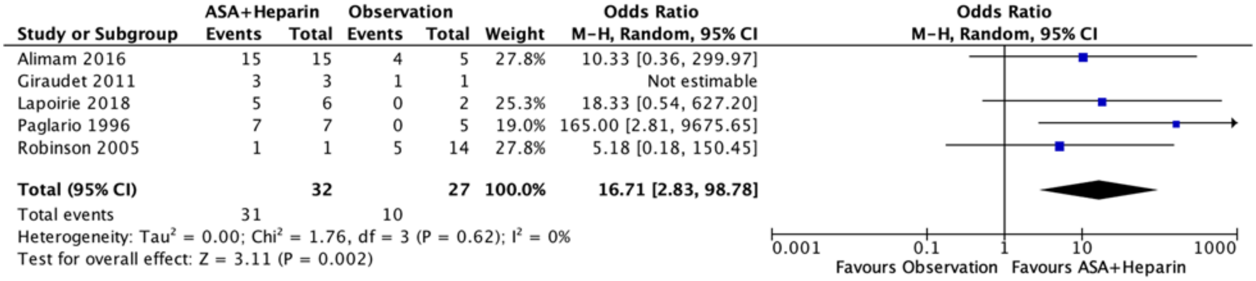

eFigure 1. Live births in patients managed with ASA + heparin vs. observation

Study or Subgroup	ASA+Heparin Events	ASA+Heparin Total	Heparin Events	Heparin Total	Weight	Odds Ratio M–H, Random, 95% CI
Alimam 2016	15	15	3	3		Not estimable
Giona 2012	3	3	2	2		Not estimable
Lapoirie 2018	5	6	2	2	100.0%	0.73 [0.02, 25.09]
**Total (95% CI)**		**24**		**7**	**100.0%**	**0.73 [0.02, 25.09]**
Total events	23		7			

Heterogeneity: Not applicable
Test for overall effect: Z = 0.17 (P = 0.86)

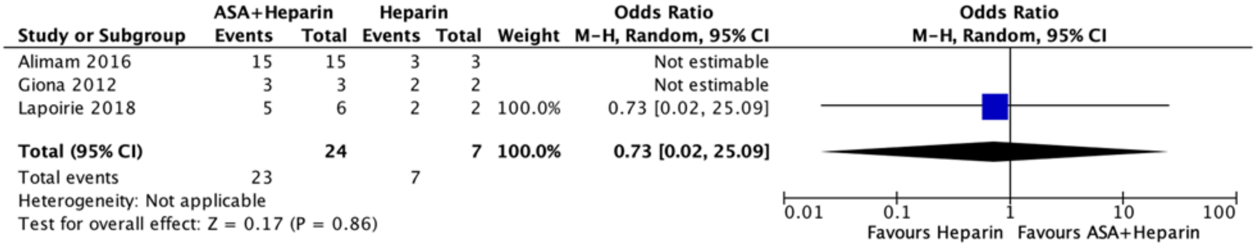

eFigure 2. Live births in patients managed with ASA + heparin vs. heparin

Study or Subgroup	Heparin Events	Heparin Total	Observation Events	Observation Total	Weight	Odds Ratio M–H, Random, 95% CI
Alimam 2016	3	3	4	5	60.2%	2.33 [0.07, 76.67]
Lapoirie 2018	2	2	0	2	39.8%	25.00 [0.34, 1831.59]
**Total (95% CI)**		**5**		**7**	**100.0%**	**6.00 [0.40, 90.08]**
Total events	5		4			

Heterogeneity: Not applicable :hi² = 0.71, df = 1 (P = 0.40); I² = 0%
Test for overall effect: Z = 1.40 (P = 0.20)

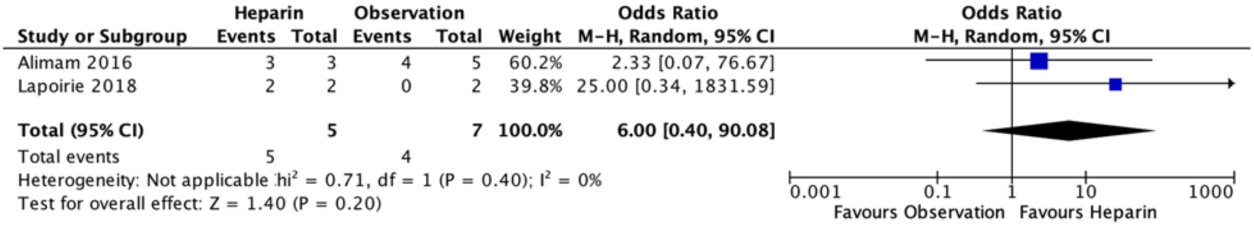

eFigure 3. Live births in patients managed with heparin vs. observation

Study or Subgroup	IFN Events	IFN Total	No IFN Events	No IFN Total	Weight	Odds Ratio M–H, Random, 95% CI
Cincotta 2000	4	4	13	26	14.3%	9.00 [0.44, 183.97]
Giraudet 2011	3	3	13	15	12.3%	1.30 [0.05, 33.63]
Lapoirie 2018	4	6	16	21	33.5%	0.63 [0.09, 4.49]
Nittyvuopio 2004	2	2	23	38	13.5%	3.30 [0.15, 73.45]
Puyade 2017	5	5	8	10	12.6%	3.24 [0.13, 80.99]
Robinson 2005	3	3	12	22	13.8%	5.88 [0.27, 127.26]
**Total (95% CI)**		**23**		**132**	**100.0%**	**2.10 [0.67, 6.58]**
Total events	21		85			

Heterogeneity: Tau² = 0.00; Chi² = 3.09, df = 5 (P = 0.69); I² = 0%
Test for overall effect: Z = 1.27 (P = 0.20)

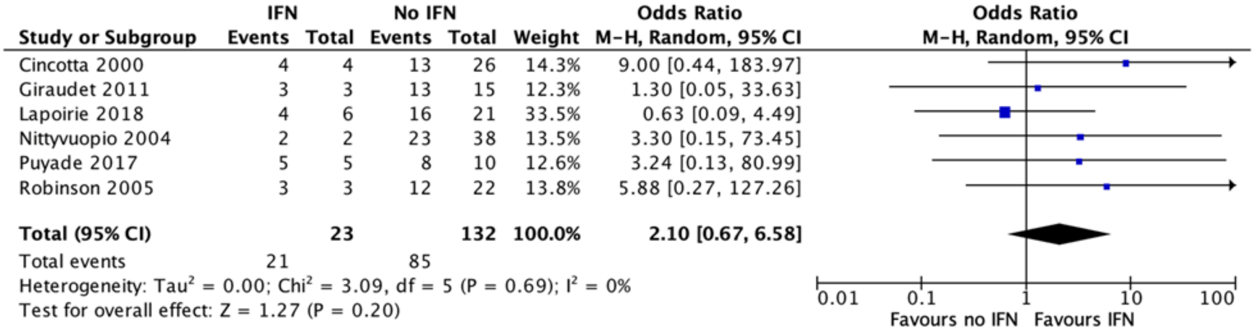

eFigure 4. Live births in patients managed with IFN vs. no IFN (with or without other interventions)

© 2019 Maze D et al. *JAMA Network Open.*

Study or Subgroup	IFN Events	IFN Total	Observation Events	Observation Total	Weight	Odds Ratio M-H, Random, 95% CI
Cincotta 2000	3	3	6	16	100.0%	11.31 [0.50, 256.20]
**Total (95% CI)**		**3**		**16**	**100.0%**	**11.31 [0.50, 256.20]**
Total events	3		6			

Heterogeneity: Not applicable
Test for overall effect: Z = 1.52 (P = 0.13)

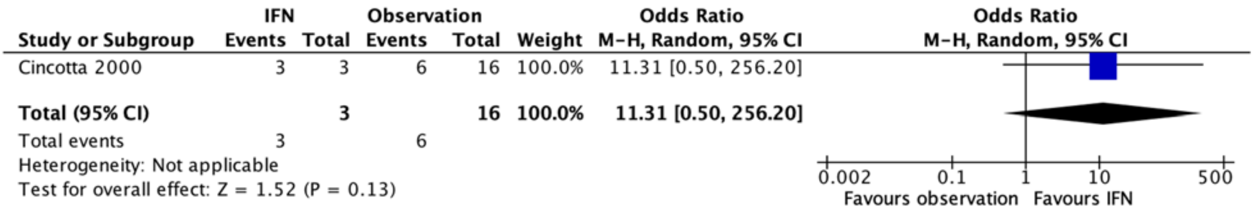

eFigure 5. Live births in patients managed with IFN vs. observation

Study or Subgroup	INF+ASA Events	INF+ASA Total	ASA Events	ASA Total	Weight	Odds Ratio M-H, Random, 95% CI
Giraudet 2011	2	2	9	11	54.6%	1.32 [0.05, 37.16]
Nittyvuopio 2004	2	2	8	8		Not estimable
Puyade 2017	4	4	6	6		Not estimable
Robinson 2005	1	1	6	7	45.4%	0.69 [0.02, 26.90]
**Total (95% CI)**		**9**		**32**	**100.0%**	**0.98 [0.08, 11.59]**
Total events	9		29			

Heterogeneity: $Tau^2 = 0.00; Chi^2 = 0.06, df = 1 (P = 0.80); I^2 = 0\%$
Test for overall effect: Z = 0.01 (P = 0.99)

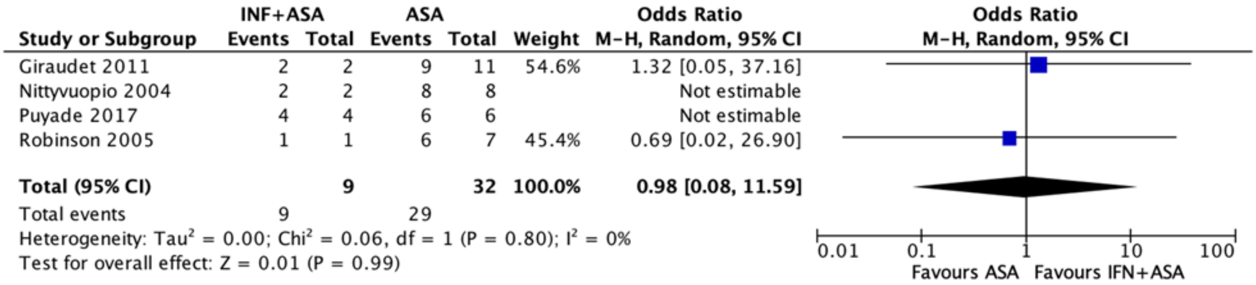

eFigure 6. Live births in patients managed with IFN + ASA vs. ASA

Study or Subgroup	IFN+ASA+Heparin Events	IFN+ASA+Heparin Total	ASA+Heparin Events	ASA+Heparin Total	Weight	Odds Ratio M-H, Random, 95% CI
Giraudet 2011	1	1	3	3		Not estimable
Lapoirie 2018	3	5	5	6	100.0%	0.30 [0.02, 4.91]
Robinson 2005	2	2	1	1		Not estimable
**Total (95% CI)**		**8**		**10**	**100.0%**	**0.30 [0.02, 4.91]**
Total events	6		9			

Heterogeneity: Not applicable
Test for overall effect: Z = 0.84 (P = 0.40)

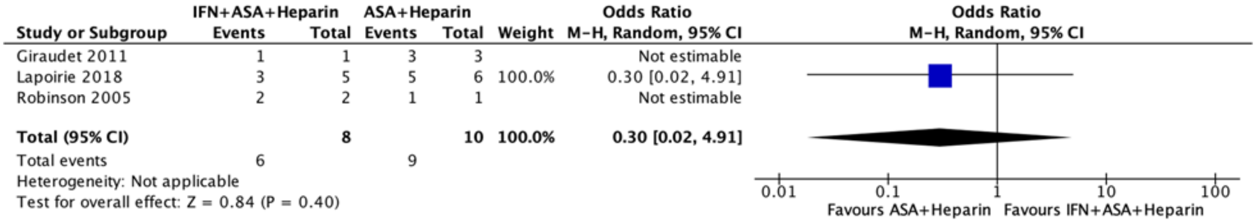

eFigure 7. Live births in patients managed with IFN + ASA + Heparin vs. ASA + Heparin

Study or Subgroup	ASA Events	ASA Total	Observation Events	Observation Total	Weight	Odds Ratio M-H, Random, 95% CI
Alimam 2016	4	25	0	7	13.8%	3.14 [0.15, 65.48]
Bangerter 2005	0	6	3	11	13.0%	0.19 [0.01, 4.29]
Betti 2015	0	14	0	7		Not estimable
Cincotta 2000	0	10	2	16	12.9%	0.28 [0.01, 6.37]
Giraudet 2011	2	11	1	1	10.5%	0.09 [0.00, 2.88]
Lapoirie 2018	3	11	0	2	11.8%	2.06 [0.08, 54.80]
Nittyvuopio 2004	0	8	3	30	13.6%	0.46 [0.02, 9.87]
Paglario 1996	3	3	3	5	11.2%	5.00 [0.17, 146.64]
Puyade 2017	0	6	0	4		Not estimable
Robinson 2005	0	7	3	14	13.2%	0.22 [0.01, 4.88]
**Total (95% CI)**		**101**		**97**	**100.0%**	**0.59 [0.19, 1.84]**
Total events	12		15			

Heterogeneity: $Tau^2 = 0.00; Chi^2 = 5.57, df = 7 (P = 0.59); I^2 = 0\%$
Test for overall effect: Z = 0.91 (P = 0.37)

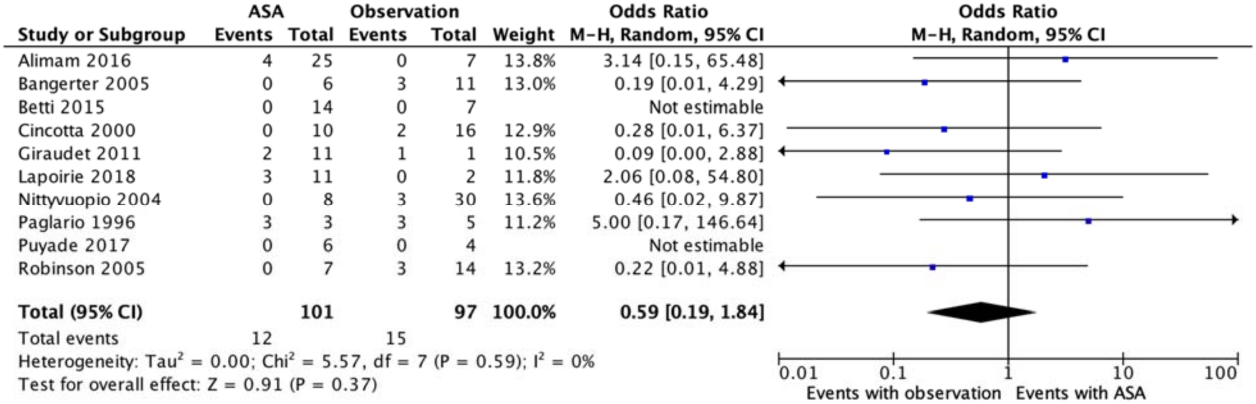

eFigure 8. Maternal events in patients managed with ASA vs. observation

© 2019 Maze D et al. *JAMA Network Open.*

Study or Subgroup	ASA+Heparin Events	ASA+Heparin Total	Observation Events	Observation Total	Weight	Odds Ratio M-H, Random, 95% CI
Alimam 2016	1	26	0	7	17.7%	0.88 [0.03, 23.98]
Betti 2015	0	32	0	7		Not estimable
Giraudet 2011	1	3	1	1	13.5%	0.20 [0.00, 8.82]
Lapoirie 2018	2	6	0	2	16.7%	2.78 [0.09, 83.84]
Paglario 1996	3	7	3	5	35.5%	0.50 [0.05, 5.15]
Robinson 2005	1	1	3	14	16.6%	9.86 [0.32, 300.41]
**Total (95% CI)**		**75**		**36**	**100.0%**	**1.07 [0.27, 4.28]**
Total events	8		7			

Heterogeneity: $Tau^2 = 0.00$; $Chi^2 = 3.10$, df = 4 (P = 0.54); $I^2 = 0\%$
Test for overall effect: Z = 0.09 (P = 0.93)

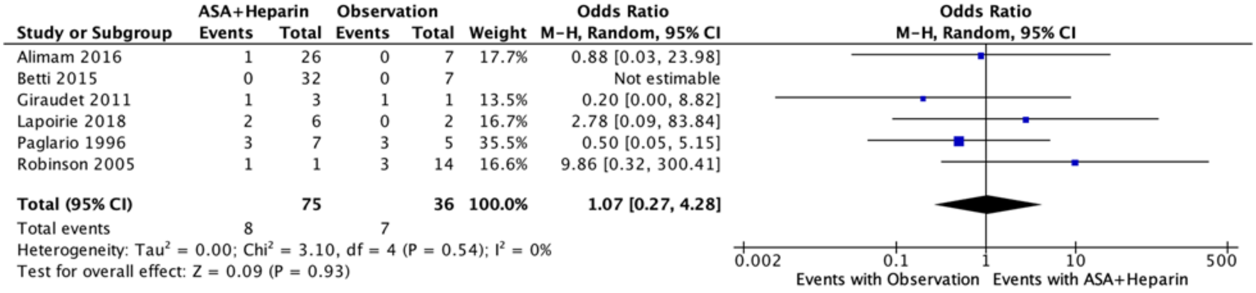

eFigure 9. Maternal events in patients managed with ASA + Heparin vs. observation

Study or Subgroup	ASA+Heparin Events	ASA+Heparin Total	ASA Events	ASA Total	Weight	Odds Ratio M-H, Random, 95% CI
Alimam 2016	1	26	4	25	25.4%	0.21 [0.02, 2.03]
Betti 2015	0	32	0	14		Not estimable
Giraudet 2011	1	3	2	11	19.9%	2.25 [0.13, 38.81]
Lapoirie 2018	2	6	3	11	26.7%	1.33 [0.15, 11.50]
Paglario 1996	3	7	3	3	16.7%	0.11 [0.00, 2.94]
Robinson 2005	1	1	0	7	11.3%	45.00 [0.61, 3296.87]
**Total (95% CI)**		**75**		**71**	**100.0%**	**0.91 [0.18, 4.71]**
Total events	8		12			

Heterogeneity: $Tau^2 = 1.43$; $Chi^2 = 6.85$, df = 4 (P = 0.14); $I^2 = 42\%$
Test for overall effect: Z = 0.11 (P = 0.91)

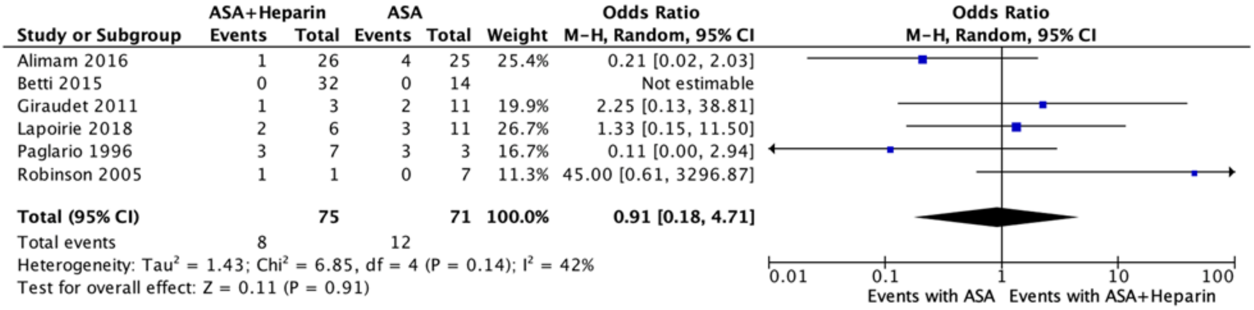

eFigure 10. Maternal events in patients managed with ASA + Heparin vs. ASA

Study or Subgroup	ASA+Heparin Events	ASA+Heparin Total	Heparin Events	Heparin Total	Weight	Odds Ratio M-H, Random, 95% CI
Betti 2015	0	32	0	6		Not estimable
Lapoirie 2018	1	6	2	2	100.0%	0.05 [0.00, 1.87]
**Total (95% CI)**		**6**		**2**	**100.0%**	**0.05 [0.00, 1.87]**
Total events	1		2			

Heterogeneity: Not applicable
Test for overall effect: Z = 1.61 (P = 0.11)

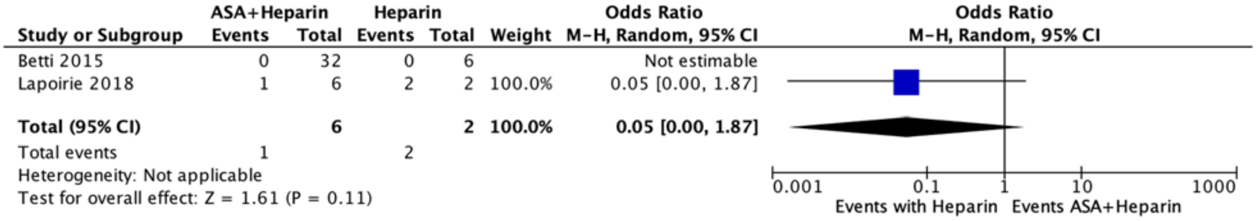

eFigure 11. Maternal events in patients managed with ASA + heparin vs. heparin

Study or Subgroup	Heparin Events	Heparin Total	Observation Events	Observation Total	Weight	Odds Ratio M-H, Random, 95% CI
Betti 2015	0	6	0	7		Not estimable
Lapoirie 2018	2	2	0	2	100.0%	25.00 [0.34, 1831.59]
**Total (95% CI)**		**8**		**9**	**100.0%**	**25.00 [0.34, 1831.59]**
Total events	2		0			

Heterogeneity: Not applicable
Test for overall effect: Z = 1.47 (P = 0.14)

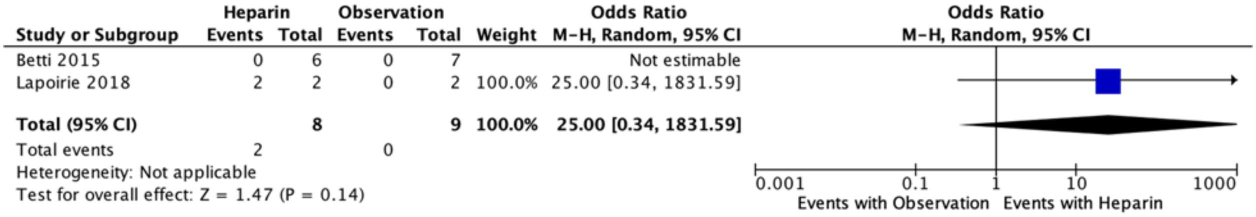

eFigure 12. Maternal events in patients managed with heparin vs. observation

© 2019 Maze D et al. *JAMA Network Open.*

Study or Subgroup	IFN Events	IFN Total	Observation Events	Observation Total	Weight	Odds Ratio M–H, Random, 95% CI
Alimam 2016	0	8	0	7		Not estimable
Betti 2015	0	9	0	7		Not estimable
Cincotta 2000	0	4	2	16	18.3%	0.64 [0.03, 16.05]
Giraudet 2011	1	3	1	1	13.2%	0.20 [0.00, 8.82]
Lapoirie 2018	2	6	0	2	16.3%	2.78 [0.09, 83.84]
Nittyvuopio 2004	0	2	3	30	18.0%	1.57 [0.06, 39.87]
Puyade 2017	1	5	0	4	15.8%	3.00 [0.09, 95.17]
Robinson 2005	0	3	3	14	18.5%	0.47 [0.02, 11.47]
**Total (95% CI)**		**40**		**81**	**100.0%**	**0.99 [0.25, 3.91]**
Total events	4		9			

Heterogeneity: $Tau^2 = 0.00$; $Chi^2 = 1.79$, df = 5 (P = 0.88); $I^2 = 0\%$

Test for overall effect: Z = 0.02 (P = 0.99)

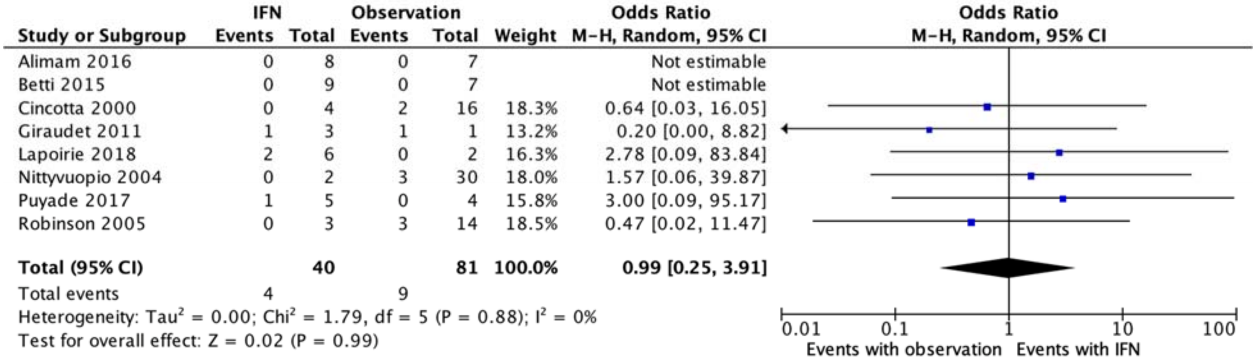

eFigure 13. Maternal events in patients managed with IFN (with or without other interventions) vs. observation

Study or Subgroup	IFN Events	IFN Total	No IFN Events	No IFN Total	Weight	Odds Ratio M–H, Random, 95% CI
Cincotta 2000	0	4	2	26	12.4%	1.09 [0.04, 26.67]
Giraudet 2011	1	3	4	15	17.9%	1.38 [0.10, 19.64]
Lapoirie 2018	2	6	6	21	33.6%	1.25 [0.18, 8.73]
Nittyvuopio 2004	0	2	3	38	12.2%	2.03 [0.08, 51.22]
Puyade 2017	1	5	0	10	11.1%	7.00 [0.24, 206.78]
Robinson 2005	0	3	4	22	12.9%	0.59 [0.03, 13.53]
**Total (95% CI)**		**23**		**132**	**100.0%**	**1.46 [0.47, 4.49]**
Total events	4		19			

Heterogeneity: $Tau^2 = 0.00$; $Chi^2 = 1.25$, df = 5 (P = 0.94); $I^2 = 0\%$

Test for overall effect: Z = 0.65 (P = 0.51)

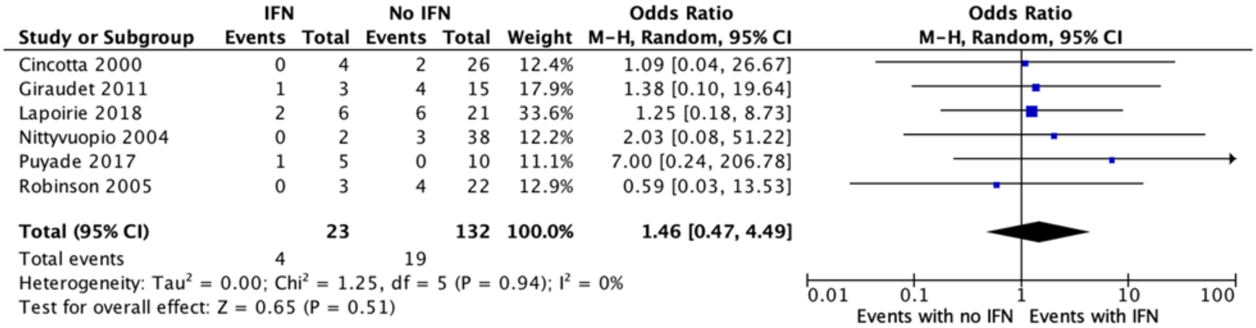

eFigure 14. Maternal events in patients managed with IFN vs. no IFN (with or without other interventions)

Study or Subgroup	IFN Events	IFN Total	Observation Events	Observation Total	Weight	Odds Ratio M–H, Random, 95% CI
Cincotta 2000	0	3	2	16	100.0%	0.83 [0.03, 21.43]
**Total (95% CI)**		**3**		**16**	**100.0%**	**0.83 [0.03, 21.43]**
Total events	0		2			

Heterogeneity: Not applicable

Test for overall effect: Z = 0.11 (P = 0.91)

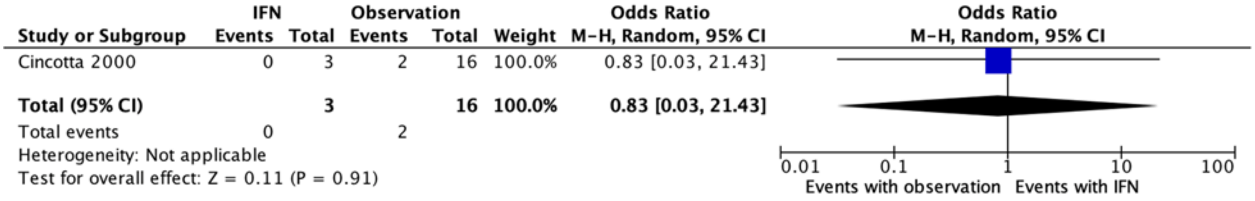

eFigure 15. Maternal events in patients managed with IFN vs. observation

Study or Subgroup	INF+ASA Events	INF+ASA Total	ASA Events	ASA Total	Weight	Odds Ratio M–H, Random, 95% CI
Giraudet 2011	1	2	2	11	54.3%	4.50 [0.19, 106.82]
Nittyvuopio 2004	0	2	0	8		Not estimable
Puyade 2017	1	4	0	6	45.7%	5.57 [0.18, 176.26]
Robinson 2005	0	1	0	7		Not estimable
**Total (95% CI)**		**9**		**32**	**100.0%**	**4.96 [0.48, 51.22]**
Total events	2		2			

Heterogeneity: $Tau^2 = 0.00$; $Chi^2 = 0.01$, df = 1 (P = 0.93); $I^2 = 0\%$

Test for overall effect: Z = 1.34 (P = 0.18)

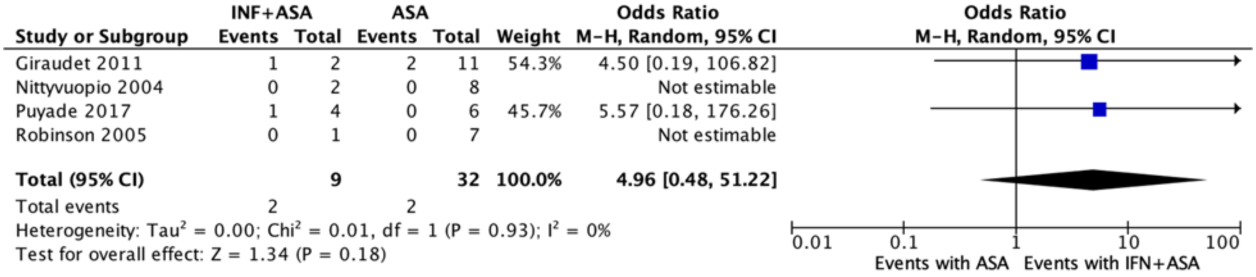

eFigure 16. Maternal events in patients managed with IFN + ASA vs. ASA

© 2019 Maze D et al. *JAMA Network Open.*

Study or Subgroup	IFN+ASA+Heparin Events	Total	ASA+Heparin Events	Total	Weight	Odds Ratio M-H, Random, 95% CI
Giraudet 2011	0	1	1	3	27.8%	0.56 [0.01, 24.51]
Lapoirie 2018	1	5	2	6	51.8%	0.50 [0.03, 7.99]
Robinson 2005	0	2	1	1	20.4%	0.07 [0.00, 5.49]
**Total (95% CI)**		**8**		**10**	**100.0%**	**0.34 [0.05, 2.51]**
Total events	1		4			

Heterogeneity: Tau² = 0.00; Chi² = 0.66, df = 2 (P = 0.72); I² = 0%
Test for overall effect: Z = 1.06 (P = 0.29)

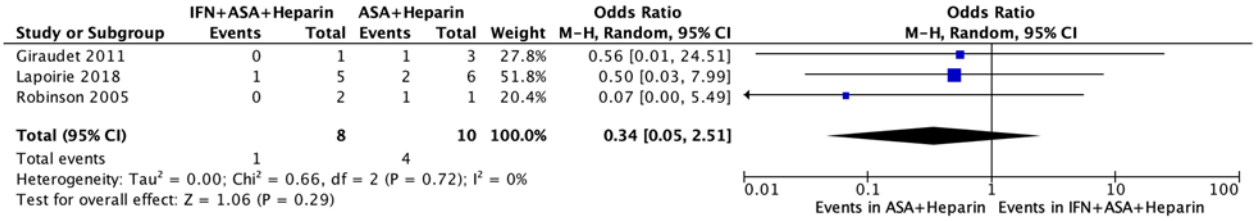

eFigure 17. Maternal events in patients managed with IFN + ASA + Heparin vs. ASA + Heparin

Study or Subgroup	Postpartum heparin Events	Total	No postpartum heparin Events	Total	Weight	Odds Ratio M-H, Random, 95% CI
Bangerter 2005	0	5	3	12	30.7%	0.25 [0.01, 5.72]
Betti 2015	0	45	1	6	28.5%	0.04 [0.00, 1.12]
Lapoirie 2018	1	5	3	22	40.8%	1.58 [0.13, 19.42]
Robinson 2005	0	11	0	14		Not estimable
**Total (95% CI)**		**55**		**40**	**100.0%**	**0.31 [0.04, 2.61]**
Total events	1		7			

Heterogeneity: Tau² = 1.23; Chi² = 3.07, df = 2 (P = 0.22); I² = 35%
Test for overall effect: Z = 1.07 (P = 0.28)

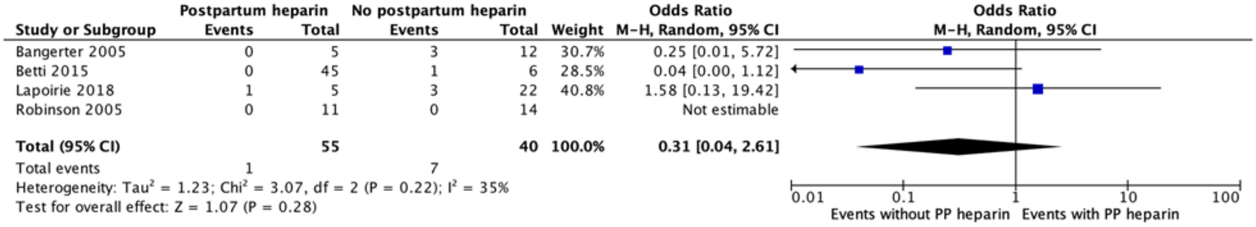

eFigure 18. Maternal events in patients managed with postpartum heparin vs. no postpartum heparin

© 2019 Maze D et al. *JAMA Network Open.*

eTable 4: Quality of evidence for live birth rates and maternal adverse events

ASA compared to Observation

Outcomes	No of Participants (studies)	Quality of the evidence (GRADE)	Relative effect (95% CI)	Anticipated absolute effects	
				Risk with Observation	Risk difference with ASA (95% CI)
**Live birth rate**	227 (11 studies)	⊕⊕⊖⊖ **MODERATE**[1] due to large effect	**OR 8.55** (4.03 to 18.12)	**39 per 100**	**45 more per 100** (from 33 more to 53 more)
**Maternal outcomes**	167 (8 studies)	⊕⊖⊖⊖ **VERY LOW**[2-6] due to risk of bias, inconsistency, imprecision	**OR 0.59** (0.19 to 1.84)	**17 per 100**	**6 fewer per 100** (from 14 fewer to 11 more)

Interferon in addition to other interventions compared to Observation

Outcomes	No of Participants (studies)	Quality of the evidence (GRADE)	Relative effect (95% CI)	Anticipated absolute effects	
				Risk with Observation	Risk difference with Interferon in addition to other interventions (95% CI)
**Live birth rate**	86 (5 studies)	⊕⊕⊕⊖ **MODERATE**[1] due to large effect	**OR 9.72** (2.31 to 41.01)	**42 per 100**	**45 more per 100** (from 21 more to 54 more)
**Maternal outcomes**	94 (6 studies)	⊕⊖⊖⊖ **VERY LOW**[6,7] due to risk of bias, inconsistency, imprecision	**OR 0.99** (0.25 to 3.91)	**13 per 100**	**0 fewer per 100** (from 10 fewer to 24 more)

ASA and heparin compared to Observation

Outcomes	No of Participants (studies)	Quality of the evidence (GRADE)	Relative effect (95% CI)	Anticipated absolute effects	
				Risk with Control	Risk difference with ASA and heparin compared to observation (95% CI)
**Live birth rate**	56 (5 studies)	⊕⊖⊖⊖ **LOW**[1] due to imprecision, large effect	**OR 15.75** (2.66 to 93.25)	**37 per 100**	**53 more per 100** (from 24 more to 61 more)
**Maternal outcomes**	72 (5 studies)	⊕⊖⊖⊖ **VERY LOW**[8,9] due to risk of bias, inconsistency, imprecision	**OR 1.07** (0.27 to 4.28)	**24 per 100**	**1 more per 100** (from 16 fewer to 34 more)

ASA and heparin compared to ASA

Outcomes	No of Participants (studies) Follow up	Quality of the evidence (GRADE)	Relative effect (95% CI)	Anticipated absolute effects	
				Risk with ASA	Risk difference with ASA and heparin (95% CI)

© 2019 Maze D et al. *JAMA Network Open.*

**Live birth rate**	57<br>(5 studies)	⊕⊖⊖⊖<br>**VERY LOW**[8]<br>due to risk of bias, inconsistency, imprecision, plausible confounding would change the effect	**OR 2.13**<br>(0.5 to 9.03)	**78 per 100**	**10 more per 100**<br>(from 14 fewer to 19 more)
**Maternal outcomes**	100<br>(5 studies)	⊕⊖⊖⊖<br>**VERY LOW**[8,9]<br>due to risk of bias, inconsistency, imprecision, plausible confounding would change the effect	**OR 0.91**<br>(0.18 to 4.71)	**21 per 100**	**2 fewer per 100**<br>(from 16 fewer to 35 more)

Heparin compared to Observation

**Outcomes**	**No of Participants (studies)**	**Quality of the evidence** (GRADE)	**Relative effect (95% CI)**	**Anticipated absolute effects**	
				**Risk with Observation**	**Risk difference with Heparin** (95% CI)
**Live birth rate**	12<br>(2 studies)	⊕⊖⊖⊖<br>**VERY LOW**<br>due to risk of bias, imprecision	**OR 6**<br>(0.4 to 90.08)	**57 per 100**	**32 more per 100**<br>(from 22 fewer to 42 more)

[1] The results were consistent across studies.
[2] The relative risk is > 2.
[3] The dose, timing and previous pregnancy outcomes were not specified in many studies.
[4] Hemorrhage was inconsistently reported
[5] Prior history of thrombosis or hemorrhage may have affected outcomes
[6] The use of ASA may have affected the rate of preeclampsia which was often not reported.
[7] The event may have preceded the pregnancy.
[8] Results were inconsistent across studies.
[9] Outcomes were not systematically assessed.
[10] Relative risk is <0.5.
[11] Potential confounding variables could affect the risk estimate.

© 2019 Maze D et al. *JAMA Network Open.*
